# Impact of Behavioral Assessment and Re-Test as Functional Trainings That Modify Survival, Anxiety and Functional Profile (Physical Endurance and Motor Learning) of Old Male and Female 3xTg-AD Mice and NTg Mice with Normal Aging

**DOI:** 10.3390/biomedicines10050973

**Published:** 2022-04-22

**Authors:** Lidia Castillo-Mariqueo, Lydia Giménez-Llort

**Affiliations:** 1 Institut de Neurociències, Universitat Autònoma de Barcelona, 08193 Barcelona, Spain; lidia.castillom@autonoma.cat; 2 Department of Psychiatry and Forensic Medicine, School of Medicine, Universitat Autònoma de Barcelona, 08193 Barcelona, Spain

**Keywords:** Alzheimer’s disease, aging, survival, anxious profile, functional profile, motor performance, frailty, training, gait, kyphosis

## Abstract

Longitudinal approaches for disease-monitoring in old animals face survival and frailty limitations, but also assessment and re-test bias on genotype and sex effects. The present work investigated these effects on 56 variables for behavior, functional profile, and biological status of male and female 3xTg-AD mice and NTg counterparts using two designs: (1) a longitudinal design: naïve 12-month-old mice re-tested four months later; and (2) a cross-sectional design: naïve 16-month-old mice compared to those re-tested. The results confirmed the impact as (1) improvement of survival (NTg rested females), variability of gait (3xTg-AD 16-month-old re-tested and naïve females), physical endurance (3xTg-AD re-tested females), motor learning (3xTg-AD and NTg 16-month-old re-tested females), and geotaxis (3xTg-AD naïve 16-month-old males); but (2) worse anxiety (3xTg-AD 16-month-old re-tested males), HPA axis (3xTg-AD 16-month-old re-tested and naïve females) and sarcopenia (3xTg-AD 16-month-old naïve females). Males showed more functional correlations than females. The functional profile, biological status, and their correlation are discussed as relevant elements for AD-pathology. Therefore, repetition of behavioral batteries could be considered training by itself, with some variables sensitive to genotype, sex, and re-test. In the AD-genotype, females achieved the best performance in physical endurance and motor learning, while males showed a deterioration in most studied variables.

## 1. Introduction

Specific motor skills impaired in old age include a broad and varied spectrum that involves a reduction in gait speed, loss of strength and muscle mass, and decline of balance [1,2,3]. However, aging has become increasingly recognized as a potentially modifiable risk factor for chronic disease and frailty [4,5]. The deterioration of motor performance related to cognitive dysfunction in Alzheimer’s disease (AD) has recently gained importance in clinical research [6,7,8,9]. Particularly, gait impairment and its association with cognitive impairment [10] could shed light on potentialities to distinguish AD [1]. Inclusive, higher levels of Aβ and tau are associated with more significant memory decline, but not with changes in executive function [11]. The study by Sperling points out that these results could explain why some clinically active patients presented elevated tau and Aβ levels [11]. Thus, Aβ and tau proteins can serve as markers of cognitive impairment; however, they are insufficient and cannot detect all cases of dementia, especially in the early stages [11,12]. For this part, gait speed, for example, is longitudinally associated with cognitive decline, dementia, and falls in older adults [13,14], with slower gait associated with increased fall risk and poor baseline cognition [6]. However, motor dysfunctions and deterioration remain poorly explored. Consequently, functional and cognitive decline comorbidity is a warning sign for increased disability [8], a growing public health problem [15], and it is already present at preclinical stages of Alzheimer’s disease [5].

On the other hand, aging is a frequent risk factor for different diseases, including dementia [16]. Recently, in a review of the literature that examined the pathophysiological basis and biomarkers of AD and other neurodegenerative diseases, it was pointed out that the predisposing factors for neuroinflammation are aging, metabolic diseases, hypertension, cerebrovascular accidents, depression and depression, dementia, among others [17]. In addition, healthy aging would be associated with chronic inflammation, contributing to a greater vulnerability to anxiety and depression [17]. Thus, cause–effect relationships can become bidirectional in the pathogenesis of multifactorial diseases, leading to a disease-prone state [18]. Age-related deficits in the ability to process contextual information and regulate responses to threat, addressing that structural and physiological alterations in the prefrontal cortex and medial temporal lobe determine cognitive changes in advanced aging, which may eventually cause patterns of cognitive dysfunction seen in patients with AD and mild cognitive impairment (MCI) [19].

Furthermore, it is known that AD is characterized by high heterogeneity in the disease’s manifestation, progression, and risk factors [20]. Such a high phenotypic variability is considered one of the most significant obstacles in early diagnosis and clinical trial design [20]. Therefore, there is great interest in identifying factors driving variability used for patient stratification [20,21]. Additionally, the impact of sex on the disease varies throughout its progression [22,23]. It is important to identify the role of sex differences in the cognitive dimension if potentially more precise diagnoses and treatments should emerge [24,25], but few studies have reported differences in the psychomotor functional dimension of the disease.

In the last decade, at the translational level, the impact of interventions on age-related disability, frailty, and the onset of AD has been investigated in animal models to develop clinically relevant measures that provide indications for the approach and management of disability, frailty, and illness [26,27]. In addition, genotype and sex differences in cognitive, emotional, and locomotor performance have been studied at the preclinical level to assess the effects of promising interventions before their application in clinical settings [28,29,30].

Recently, our laboratory has developed a study method to identify psychomotor impairments and deficits at different stages of Alzheimer’s disease [31]. Previously, we reported a functional impairment phenotype in male mice’s gait and physical performance of the 3xTg-AD transgenic model in the initial, intermediate, and advanced stages of the disease. The results showed that 3xTg-AD mice show a significant functional impairment in the quantitative variables of gait and exploratory activity, movement limitations, and muscle weakness related to functional decline in the different stages of severity of the disease intensify with increasing age. In addition, signs of frailty accompany functional deterioration, and sarcopenia is evident in an advanced stage of AD, with differences in the morphological characteristics of muscle fibers and the number of fat cells [31].

Furthermore, we differentiate the disorders and postural patterns into two types of kyphosis (postural and structural) that differ in severity and limit the exploratory activity. In addition, the results indicated that the presence of bizarre gait patterns accounts for behavior similar to anxiety when 3xTg-AD mice face novelty situations and recognition of places, with circling and backward movements being the most frequent, in an already frailty setting [32,33].

The present study was designed to investigate the impact of two factors, sex, and repeated test, assessing the behavioral outputs of 3xTg-AD mice and mice with normal aging in longitudinal and transversal experimental designs. According to our previous work, a battery of psychomotor tests: gait, exploration, muscle strength, motor learning, physical endurance, and frailty status, was used [31]. In addition, phenotype of frailty and biological status (HPA axis and sarcopenia index) was also included [32,33]. Thus, in the first place, we studied the sex factor by characterizing the psychomotor phenotype of middle—(12 months) and old—(16 months) age females, that in the 3xTg-AD mice corresponds to two neuropathological stages of the disease [31], as compared to that of aged-matched males. On the other hand, long-term studies provide better insights for assessing interventions with preclinical validity, but the administration of behavioral batteries is not exempt from carryover effects. In addition, behavioral batteries and repeated tests can be considered behavioral stimulation [34]. Therefore, we aimed to investigate the effects of repeated tests on the behavioral performance of animals assessed in two scenarios: 1) in a longitudinal design, with within-subjects analysis of a set of 12-month-old animals re-tested four months later, at 16 months of age; and 2) in a transversal design, when comparing 16-month-old animals that had (re-tested) or not (naïve) that experienced the battery of tests. 

## 2. Materials and Methods

### 2.1. Animals

A total of 191 male and female mice were included for the survival analysis, and ninety-six of them, homozygous 3xTg-AD (*n* = 54) and non-transgenic (NTg, *n* = 42) male and female mice of 12 to 16 months of age in a C57BL/6J background (after embryo transfer and backcrossing of at least ten generations), established at the Universitat Autònoma de Barcelona, were included in the experimental study. As previously described, the 3xTg-AD mice harboring transgenes were genetically created at the University of California at Irvine [35]. Animals were kept in groups of 3–4 mice per cage (Macrolon, 35 cm×35 cm×25 cm) filled with 5 cm of clean wood cuttings (Ecopure, Chips, Date Sand, UK; uniform cross-sectional wood granules with 2.8–1.0 mm chip size) and nesting materials (Kleenex, Art: 08834060, 21 cm×20 cm, White). In all cases, standard home cages covered with a metal grid to allow the perception of olfactory and auditory stimuli from the rest of the colony. All animals were kept under standard laboratory conditions of food and water ad libitum, 20 ± 2 °C, 12 h light cycle:dark with lights turned on at 8:00 a.m. and 50–60% relative humidity. The study complied with the ARRIVE guidelines developed by the NC3Rs and aimed to reduce the number of animals used [36].

### 2.2. Experimental Design

**Figure 1:**
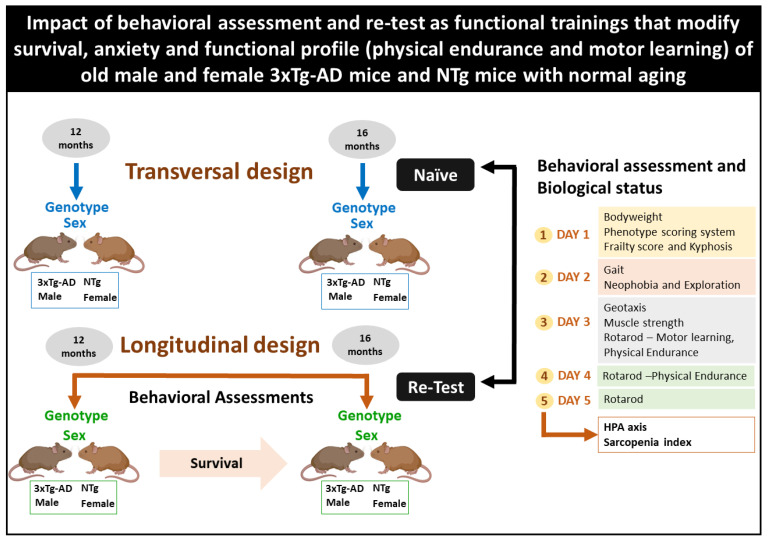
Experimental design. Longitudinal design: the first group was assessed in the behavioral battery at the age of 12 months and again when the animals reached 16 months of age. Transversal design: the second group was housed in standard conditions without manipulation until they were tested at 16 months of age, so they could be compared to re-tested 16 months old animals.

A longitudinal and a transversal study were carried out to evaluate the anxious-like and functional profiles of male and female 3xTg-AD and NTg mice. Biological variables (corticosterone and sarcopenia) of animals at the end point (16 months of age) were also included. For this purpose, animals were randomly assigned into two experimental batches (see Figure 1, Experimental design)

### 2.3. Behavioral Assessment and Biological Status

The assessment consisted of four consecutive evaluation steps conducted during 5 days, as follows: Day 1, bodyweight, phenotype scoring system, and frailty; Day 2, gait and exploration; Day 3, geotaxis, muscle strength, and rotarod; Days 4–5, rotarod. The procedures and protocol were based on the protocol used by Castillo-Mariqueo and Giménez-Llort [31]. Assessments were performed under dim white light (20 lx) in the light cycle (10:00 a.m. to 1:00 p.m.). Behavioral evaluations were carried out in a counterbalanced way by two independent observers, blind to the genotype. Animals were habituated to the test room 30 min before the start of the tests.

#### 2.3.1. Survival, Bodyweight, Phenotype Scoring System, Frailty Score, and Kyphosis

Survival curves were analyzed considering the cohort of siblings from the same litter of mice included in the study, from birth to 16 months of age. A total of 191 male and female mice, NTg and 3xTg-AD were included in this analysis (NTg males = 49; NTg females = 58; 3xTg-AD males = 50; 3xTg-AD females = 34). All animals were weighed and evaluated with the phenotype scoring system that includes four subtests and scores: ledge, grip, gait, and kyphosis [37,38]. Individual measures were scored from 0 (the absence of the relevant phenotype) to 3 (the most severe manifestation) [37]. The measures can be analyzed individually or combined into a composite phenotype score [39,40]. On the other hand, frailty was assessed using an adaptation of the MCFI by Whitehead [41], which includes 30 assessment items from the clinical setting. The 12 elements with the highest incidence previously reported by our laboratory were selected [42]. Their incidence was reported through an absence (0), presence (1) score. The clinical evaluation included physical aspects, injuries and wounds, alopecia, piloerection, body and tail position, tremor, and urogenital alterations.

#### 2.3.2. Quantitative Parameters of Gait, Neophobia, and Exploration

The quantitative parameters of the gait and exploration were recorded by filming the spontaneous gait of the mice for 1 min. Later the videos were analyzed using KINOVEA 0.8.15 free software according to the Castillo-Mariqueo and Gimenez-Llort protocol [31]. Stride length, stride length variability, speed, and cadence were included according to the methodology used by Wang et al. [43]. The examination included observation of body position, limb support, and movement. In addition, neophobia (immediate fear of a new place) was assessed by means of the corner test [44] and the recording of freezing (latency of movement), the number of explorations on the horizontal axis (visited corners), the latency and number of explorations on the vertical axis (rearings).

#### 2.3.3. Muscular Strength—Hanger Test and Geotaxis

The muscle strength was measured in the forelimbs using the hanger test. Three trials were performed to observe the tendency of a mouse to instinctively grasp a rack or bar when suspended by the tail. In the first and second trials, grip strength was assessed by holding the animal with its front legs for 5 s at the height of 40 cm. In the third trial, the animal is suspended for 60 s in a single attempt to assess muscular endurance. This test allows discriminating grip strength and muscular endurance according to the suspension times used by mice [45]. A box with sawdust is placed under the animal to prevent a possible fall in each trial. The bar used is graduated in 5-cm blocks to obtain the distance covered when the animal moves through the bar. The latency and movement distance are recorded. Geotaxis was measured using a 10 cm×12cm grid. A single trial registered the time it took for the animal to reach the vertical position from an inverted position at a 90° angle on the grid. 

#### 2.3.4. Motor Performance: Learning and Physical Endurance—Rotarod

Six micro training cycles were carried out during three consecutive days with a previous learning session and psychomotor coordination. The animals were trained in the Rotarod apparatus (Ugo basile^®^, Mouse RotaRod NG) according to a training volume established in our previous research laboratory investigations [31]. An incremental intensity of 5 to 48 rpm was applied according to individual tolerance with a maximum duration of 360 s in each microcycle with a 1-min recovery between trial. 

#### 2.3.5. Biological Status: HPA Axis and Sarcopenia Index

The animals were euthanized and the muscle tissues were necropsied. Plasma from a blood sample was obtained by centrifugation and stored and −80 °C until corticosterone analysis. Corticosterone content (ng/mL) was analyzed using a commercial kit (Corticosterone EIA Immunodiagnostic Systems Ltd., Boldon, UK). Absorbance was read at 450 nm with Varioskan LUX ESW 1.00.38 (Thermo Fisher Scientific, Massachusetts, MA, USA) [42]. The weights of the quadriceps and triceps surae muscles of the right lower extremity of each animal were recorded and kept for future analysis. The sarcopenia index [46] was applied to obtain an indirect measure of sarcopenia as a biological marker of frailty. 

### 2.4. Statistics

Statistical analyses were performed using SPSS 15.0 software. Results were expressed as the mean ± standard error of the mean (SEM) for each task and trial. The variables recorded were analyzed with Student t-test, Chi-squared or Fisher’s exact test, one-way ANOVA, and multiple regression analysis (MRA). The split-plot ANOVA design with factors genotype (G), sex (S), previous experience either as a re-test (R) in the longitudinal approach or as naïve (N) in the transversal approach, were included. Their G×S, G×R, and S×N factor interactions were also studied. *Post hoc* comparisons were run with Bonferroni corrections. Pearson’s correlations were made to analyze the functional correlations with (1) corticosterone, (2) sarcopenia index, and (3) phenotype score system. The survival curve was analyzed with the Kaplan–Meier test (Log rank). In all cases, *p* < 0.05 was considered statistically significant.

## 3. Results

### 3.1. Survival, Bodyweight, Phenotype Scoring System, Frailty Score, and Kyphosis

**Figure 2:**
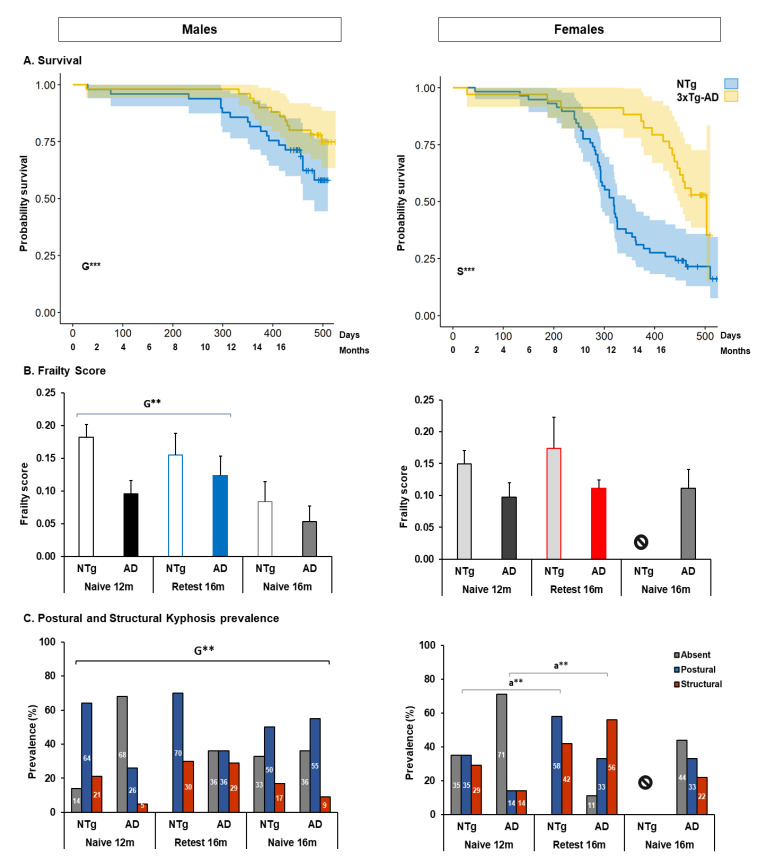
Survival, frailty score, and postural and structural kyphosis. (**A**) Survival. Statistics: Kaplan–Meier test—Log rank, G, genotype effect: χ^2^*,* G*** *p* < 0.001***; S, sex effect: χ^2^, S*** *p* < 0.001***. (**B**) Frailty score. Statistics: ANOVA, G, genotype effect: G** *p* < 0.01**. (**C**) Postural and structural kyphosis. Statistics: Fisher’s exact test, G, genotype effect in males, G** *p* < 0.01**; a, aging effect in females: a** *p* < 0.01**. The symbol ⦸ indicates the absence of the group, and m, months.

Figure 1 shows the data obtained for survival, frailty score and postural and structural kyphosis. Thus, the animal cohort was analyzed from birth to 16 months (16 months NTg and 16 months 3xTg-AD). Only siblings from the same litter belonging to mice meeting the end-points were considered. Log rank analysis showed statistically significant differences dependent on genotype and sex (G, χ^2^ (1) = 20.044, *p* < 0.001; S, χ^2^ (1) = 33.531, *p* < 0.001), see Figure 2A. In this way, it was possible to observe that females of both genotypes have higher mortality than males, and that of them the NTg is even higher (days of average survival: Males, NTg = 445.05 ± 15.75, CI: 414.16–475.94; 3xTg-AD = 505.57 ± 13.04, CI: 480.01–531.13. Females, NTg = 343.60 ± 15.39, CI: 313.42–373.78; 3xTg-AD = 442.76 ± 18.40, CI: 406.69–478.83). In addition, the female NTg cohort reached 79% (46/58) of mortality and 3xTg-AD the 50% (17/34) with ages 11 to 13 months having the greatest death. For their part, NTg males reached 37% (18/49) and 3xTg-AD 24% (12/50), with 15 to 16 months being the age of greatest death. During the follow-up of the animals that started the battery at 12 months, 20 deaths were detected, the NTg males had 29% (4/14), the 3xTg-AD males a 20% (4/20), the NTg females a 35% (7/20), and 3xTg-AD females 31% (4/13), see Figure 2A. 

In terms of frailty score, G effect were identified, the score is higher in NTg animals in naïve 12 months (frailty score, G, F (1, 62) = 11.159, *p* = 0.001), see Figure 2B. In addition, the severity of kyphosis has been identified, thus, in males, a higher prevalence of postural kyphosis has been observed, being higher in the case of NTg mice (severity, Fisher’s exact test df (15) = 24.403, *p* = 0.023. G effect, Fisher’s exact test df (3) = 11.842, *p* = 0.004). In the case of females, the highest prevalence of cases was also postural kyphosis, and this increased in 3xTg-AD mice at 16 months, being the structural type disorder in this group the one with the highest prevalence attributable to age (severity, Fisher’s exact test df (12) = 22.900, *p* = 0.008. 3xTg-AD naïve 16 months *vs.* re-test 16 months, Fisher’s exact test df (3) = 16.137, *p* = 0.001), see Figure 2C.

Table 1 shows the phenotype scoring system obtained in males and females. Specifically, at naïve 12 months, differences were detected in the gait, kyphosis, and total score, with G effect in kyphosis and total score showing the high deterioration in the NTg group (kyphosis, G, F (1, 62) = 13.329, *p* = 0.001. Total score, G, F (1, 62) = 4.078, *p* = 0.048). In addition, there is an interaction of the G×S in gait, with 3xTg-AD females and NTg males presenting a lower (gait score, G×S, F (1, 62) = 7.776, *p* = 0.007). At the 16 months re-tests, the difference in gait score is maintained without significant differences in the other parameters (gait score, G×S, F (1, 44) = 10.709, *p* = 0.002). In contrast to naïve 16 months, differences were observed in genotype and sex in the clasping score and gait, being measured the genotype differences only in males and sex between the 3xTg-AD group (clasping score, F (2, 30) = 4.646, *p* = 0.017; male 3xTg-AD naïve 16 months vs. male NTg naïve 16 months, *p* = 0.019. G, in male group, T student t = −2.836, *p* = 0.012. S, in 3xTg-AD group T student t = −2.138, *p* = 0.046). If we consider the change between the groups after the re-test, we have detected differences in the different scores of the phenotype scoring system. The main differences were detected in the total score, kyphosis and ledge score (re-test, total score, F (1, 141) = 15,972, *p* < 0.0001. Kyphosis score, F (1, 141) = 14.596, *p* < 0.000 and G, F (1, 141) = 5.159, *p* = 0.025. Ledge score, F (1, 141) = 10.435, *p* = 0.002). In addition, differences in total score, gait, kyphosis, and ledge score were detected between males, with effect of previous experience and genotype (total score, F (5, 80) = 3.449, K= 0.007, 3xTg-AD naïve 12 months vs. 3xTg-AD re-test 16 months, k = 0.031; R, F (1, 80) = 13.002, *p* = 0.001. Ledge score, R, F (1, 80) = 7.447, *p* = 0.008. Gait, F (5, 80) = 4.303, *p* = 0.002, 3xTg-AD re-test 16 months vs. 3xTg-AD naïve 16 months, *p* = 0.003; 3xTg-AD re-test 16 m vs. NTg naïve 16 months, *p* = 0.003; R, F (1, 80) = 7.461, *p* = 0.008; and G, F (1, 80) = 5.560, *p* = 0.021. Kyphosis score, F (5, 80) = 3.269, *p* = 0.010; R, F (1, 80) = 6.310, *p* = 0.014; and G, F (1, 80) = 5.225, *p* = 0.025). In females, differences were found in total score, kyphosis and gait with effect of previous experience and genotype (total score, F (4, 60) = 2,800, *p* = 0.034; R, F (1, 60) = 4.517, *p* = 0.038; G, F (1, 60) = 4.767, *p* = 0.033. Kyphosis score, F (4, 60) = 3.375, *p* = 0.015, 3xTg-AD naïve 12 months vs. 3xTg-AD re-test 16 months, *p* = 0.050; R, F (1, 60) = 7.791, *p* = 0.007. Gait score, F (4, 60) = 2.909, *p* = 0.029; G, F (1, 60) = 5.632, *p* = 0.021).

In bodyweight at 12 months was high in 3xTg-AD, and at 16 months it decreased in females. In addition, males naïve 16 months weighed more than re-test males at the same, see Tables S1–S3.

### 3.2. Quantitative Parameters of Gait, and Neophobia and Exploration

**Figure 3:**
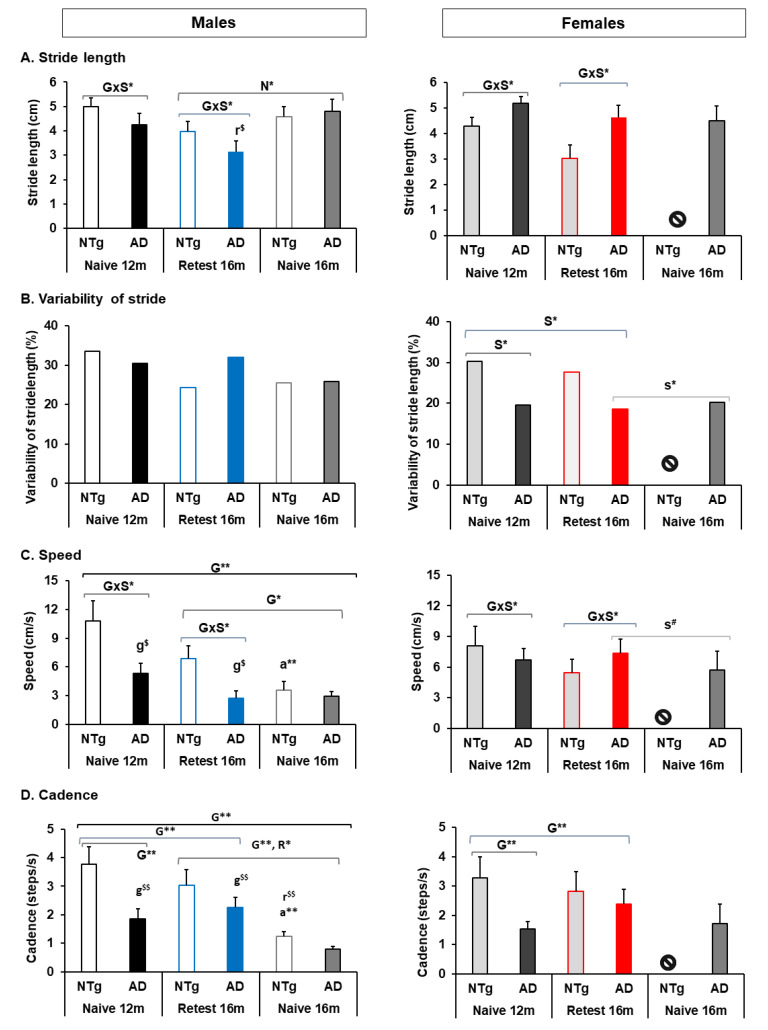
Quantitative parameters of gait. (**A**) Stride length, (**B**) variability of stride, (**C**) speed, (**D**) cadence. Statistics: ANOVA, G, genotype effect, G** *p* < 0.01**, G* *p* < 0.05*. S, sex effect, S* *p* < 0.05*. G×S, genotype and sex interaction effects, G×S* *p* < 0.05*. R, re-test effect, R* *p* < 0.05*. N, naïve effect, N* *p* < 0.05*. a, aging, a** *p* < 0.01**. Bonferroni *post hoc* test: g, genotype; s, sex; $ expressed genotype differences between sex, and # expressed sex differences between genotypes. The symbol ⦸ indicates the absence of the group, and m, month.

Quantitative parameters of gait are shown in Figure 3. For naïve 12 months, statistically significant differences were observed in all quantitative gait variables. Stride length showed differences in G and S, with the longest stride length in NTg males and 3xTg-AD in females. This interaction was also observed in gait speed, high in NTg mice. At the same time, the variability of gait presented differences associated with S, with females showing less than males’ variability and, therefore a gait with more homogeneous steps in its trajectory. Additionally, a genotype-dependent difference was observed in cadence, where NTg mice show better performance in this variable with marked differences between males. In the re-test at 16 months, this group registered a gait performance that shows the interaction between the G×S effect in stride length and speed, with the performance of 3xTg-AD females being the one with the best performance in both variables. The re-test of this group at 16 months showed differences in cadence, increasing its performance in the group of 3xTg-AD mice of both sexes and decreasing in the NTg group, see Figure 3A–D and Tables S1–S3.

On the other hand, mice at 16 months did not show significant differences in quantitative variables of gait. Differences could only be observed between the re-test and a naïve group of males at 16 months in stride length, cadence, and speed, where the re-test NTg mice presented a high performance in speed and cadence compared to the naïve NTg mice, 3xTg-AD re-test, and naïve, but lower performance in stride length than naïve mice. In addition, differences were detected between the 3xTg-AD males and females in the variable’s variability and gait speed, with the 3xTg-AD naïve and re-test females showing less variability than the 3xTg-AD males. This difference was also present in gait speed, with a better performance of re-test females followed by naïve 16 months females over 3xTg-AD males in both conditions, see Figure 3A–D and Tables S1–S3.

**Figure 4:**
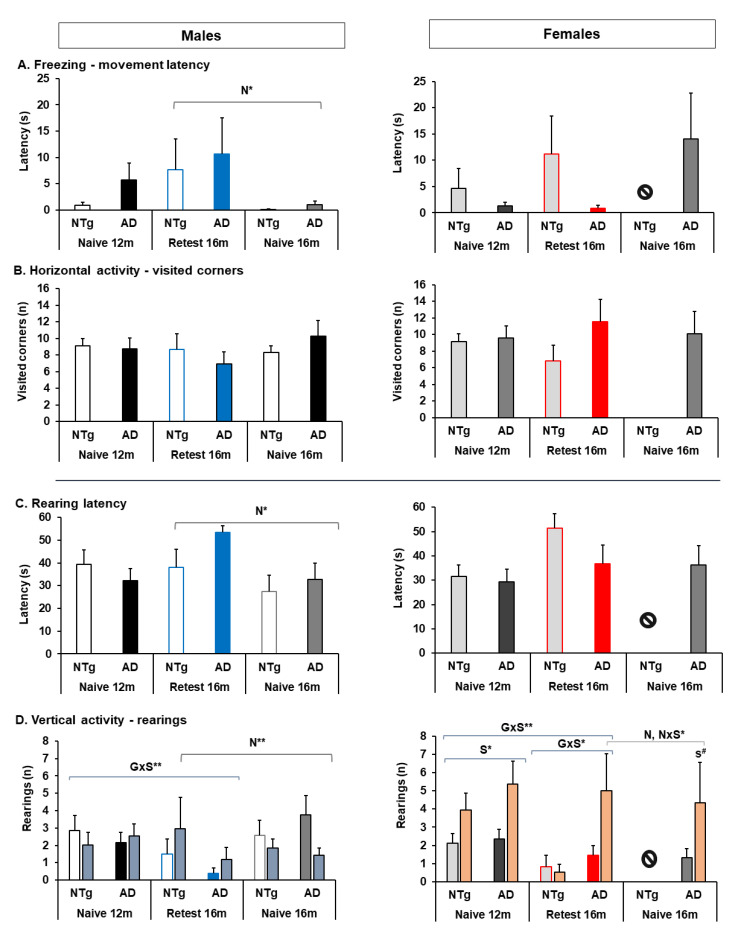
Ethogram of Neophobia and Exploratory activity. (**A**) Freezing, (**B**) horizontal activity, (**C**) rearing latency, (**D**) vertical activity. Statistics: ANOVA, S, sex effect, S* *p* < 0.05; G×S, genotype and sex interaction effects, G×S* *p* < 0.05*. N, naïve effects, naïve at 16 months *vs.* re-test 16 months, N** *p* < 0.01**, N* *p* < 0.05*. N×S, naïve and sex interaction effects, N×S* *p* < 0.05*. Bonferroni *post hoc* test: s, sex; and # expressed sex; differences between genotypes. The symbol ⦸ indicates the absence of the group, and m, month.

For its part, the neophobia and exploratory activity presented sex differences in the ratio visited corners/rearings, being higher in females of both genotypes, see Figure 4. This difference was maintained at re-test 16 months, with the higher ratio in females. In addition, the ratio in MRA of the groups showed an interaction between G×S, indicating a lower performance in 3xTg-AD re-test males at 16 months and higher in re-test females, see Figure 4B and Tables S1–S3.

As in gait, no significant differences were detected in exploratory activity between the naïve 16 months group. However, in contrast to the re-test males at 16 months, the naïve males presented high vertical activity than the re-test, see Figure 4C,D. Between the group of 3xTg-AD mice, S effect was identified in vertical activity, where naïve males presented higher activity. Movement latency was also lower in naïve males, but the same was not observed in 3xTg-AD females, see Figure 4A and Tables S1–S3.

### 3.3. Muscular Strength: Forelimb Grip Strength and Muscular Endurance—Hanger Test and Response to Gravity: Geotaxis

Lower muscle strength can be observed in the resistance distance in the 3xTg-AD animals at the age of 12 months, which, despite not showing statistical differences, shows a trend with less strength in the 3xTg-AD males. No statistically significant differences were detected in the rest of the variables, although a worse performance of the animals was observed, see Table S1. At 16 months re-test and 16 months naïve, no significant differences were detected.

**Figure 5:**
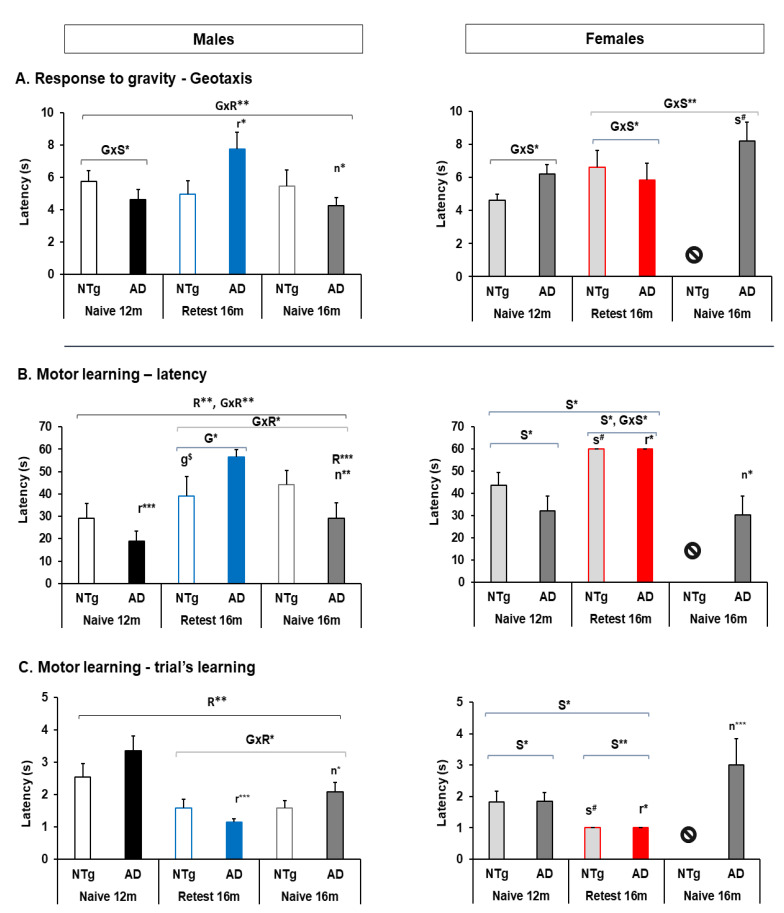
Geotaxis and motor learning—rotarod. (**A**) Geotaxis, (**B**) latency, (**C**) trial’s learning. Statistics: ANOVA, G, genotype effect, G* *p* < 0.05. S, sex effect, S** *p* < 0.01, S* *p* < 0.05. R, Re-test effect, naïve 12 months and re-test 16 months, R** *p* < 0.01**, R* *p* < 0.05*. G×S, genotype and sex interaction effects, G×S** *p* < 0.01**, G×S* *p* < 0.05*. G×R, genotype and re-test effects, G×R** *p* < 0.01**, G×R* *p* < 0.05*. Bonferroni *post hoc* test: g, genotype, s, sex, r: re-test naïve 12 months vs.16 months, and n, naïve 16 months vs. re-test 16 months; $ expressed genotype, and # expressed sex differences between genotypes. The symbol ⦸ indicates the absence of the group, and m, month.

On the other hand, significant differences in geotaxis were detected. The group of 3xTg-AD male took longer to complete the test in the re-test 16 months group in contrast to the 12 months and naïve 16 months group, see Figure 5A. Notably, at 16 months in the re-test group, an interaction was observed between the G×S of the animals, showing more significant latency in 3xTg-AD males and NTg females. G×S interaction was also observed between the group of naïve 16 months 3xTg-AD mice. In addition, there was an interaction between the G×R was detected among male mice at 16 months in contrast to naïve mice of the same age, with the test time being shorter in naïve mice, see Figure 5A and Tables S1–S3.

### 3.4. Motor Performance: Learning and Physical Endurance—Rotarod

The learning and motor performance tests in the Rotarod showed significant differences associated with different factors depending on the test or the group studied, see Figure 5. Among the males, significant differences were detected in learning and the number of trials between naïve and re-tests at 12 months and 16 months. In females, differences were detected in 3xTg-AD of 12 months and 16 months re-test and naïve, see Figure 5B,C and Tables S1–S3. In turn, for motor learning, the S effect plays an important role since females manage to learn earlier than males and spend more time on the wheel during the test at 12 months. At the re-test 16 months, the S effect was maintained in the number of trials, but in learning the G effect and G×S became important. In the same way, when performing MRA in the groups naïve at 12 months and re-test at 16 months, the S effect was the one that marked the statistical difference, see Figure 5B,C and Tables S1–S3. Nevertheless, there were no significant differences between the naïve 16 months group. However, between the re-test 16 months group vs. the naïve 16 months, differences were detected between males, where the R and G effects were significant. In addition, significant differences were also detected between the 3xTg-AD group, where the differences in S and R effects were the ones that obtained significance, see Figure 5B,C and Tables S1–S3.

**Figure 6:**
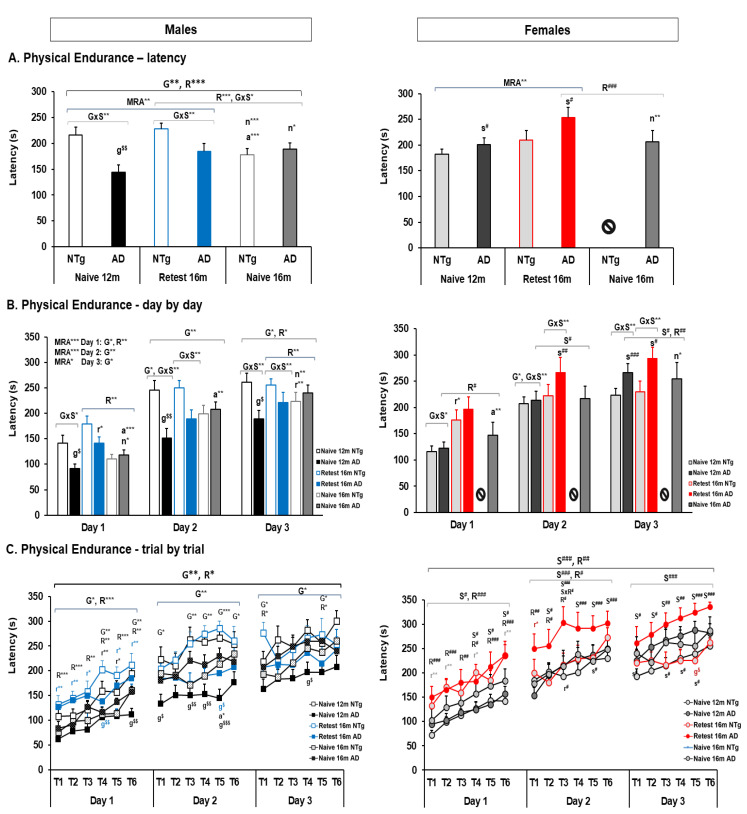
Physical endurance—rotarod. (**A**) Latency, (**B**) day by day, (**C**) trial by trial. Statistics: ANOVA, MRA-ANOVA, G, genotype effect, G** *p* < 0.01, G* *p* < 0.05. S, Sex effect, S*** *p* < 0.001***, S** *p* < 0.01**, S* *p* < 0.05. R, re-test effect, naïve 12 months vs. re-test 16 months, R*** *p* < 0.001***, R** *p* < 0.01**, R* *p* < 0.05*. G×S, genotype and sex effects, G×S** *p* < 0.01**, G×S* *p* < 0.05*. S×R, sex and re-test effects, S×R* *p* < 0.05*. Bonferroni *post hoc* test: g, genotype; s, sex; r: re-test, naïve 12 months vs. 16 months; n, naïve, naïve 16 months vs. re-test 16 months; $ expressed genotype, and # expressed sex differences between genotypes. The symbol ⦸ indicates the absence of the group, and m, month.

At the same time, it is possible to differentiate physical endurance according to the interaction of G and S in the naïve 12 months, re-test 16 months, and naïve 16 months groups, see Figure 6. The NTg males have a physical endurance similar to that of 3xTg-AD females, followed by NTg females and finally 3xTg-AD males, whose performance is low and does not improve with training. This difference persisted in the re-test at 16 months. In males, differences were also detected in the physical endurance and each training days, with significance in the age of the NTg animals and the effect of Re-test in 3xTg-AD and the NTg (see Figure 6A and Tables S1–S3). In addition, on the first day of training, it was observed that 3xTg-AD males showed differences in R and aging effect among naïve mice (see Figure 6B, and Tables S1–S3). On the second day of training, the difference in G at 12 months and the effect of aging in the naïve 3xTg-AD group stand out. The changes observed on the third day of the test were recorded at 12 months, where the G has statistical significance and the R only in NTg group. For females, physical endurance was higher in the 3xTg-AD group. The re-test 16 months group had high latencies (see Figure 6A and Table S2). Differences were observed on Day 1 and Day 3, with differences was in the 16 months re-test NTg group and aging effect in the 3xTg-AD group (see Figure 6B and Table S3). 

On the other hand, differences in genotype and sex were detected in the 12 months group (see Figure 6A and Table S1). In the 3 days of training, differences in effect were detected, with G distinction only on the second day (Figure 6B, and Table S1). Additionally, at 16 months in the re-test group, differences in G×S were recorded in physical endurance (see Figure 6A and Table S2). Days 2 and 3 showed differences in G×S, with no significant differences on the first day (see Figure 6B and Table S2). The re-test of this group corroborated the differences in G×S of the batch at 16 months (see Figure 6B and Table S2). However, at 16 months, groups of naïve mice did not show significant differences in this test. Yet, when comparing the re-test and naïve mice at 16 months, significant differences were detected between the group of males in physical endurance and the performance of Days 1 and 3 (see Figure 6A,B and Table S3). In addition, among the group of transgenic mice, differences in sex and re-test were detected between the groups, with the performance of the females being higher (see Figure 6A,B and Table S3). 

Furthermore, considering MRA between the groups, we can differentiate the effect of G, S, and R in the day-by-day and trial-by-trial tests, Table S4 shows the statistical differences from Figure 6C. The MRA analysis between males on Day 1 showed differences in G and S, see Figure 6C. It was observed that the NTg retest males improve with the repetition of the trials as well as the 3xTg-AD, but these do so to a lesser extent, and both the 12 months and 16 months naïve males have lower performance than retest. On Day 2, the genotype effect was observed here. The naïve 12 months NTg mice and the retest 16 months show a high latency in the test that increases with the execution of the trials. Naïve 16 months 3xTg-AD mice show the best performance within this group. In addition, the MRA trial-by-trial showed the differences in each trial and the animals’ G and R differences. Here, it is highlighted that the first day plays an important role in the retest and then the differences of genotype. Also, among the females, significant differences were recorded in MRA trial by trial, with the 3xTg-AD retest of 16 months being the ones with the highest performance during all the test days. On the first day of training, differences in performance were obtained between the naïve 12 m NTg females and their retest 16 months, with a higher latency between the 16 months 3xTg-AD retest (Figure 6C). The second day of training did not record differences between the females, but on the third day, the highest performance of the 3xTg-AD retest 16 months was observed again.

Additionally, it is possible to differentiate females from males in the 3xTg-AD group, with females showing better performance in all tests. Thus, on Day 1 the mice differ in S and R. On the second day, the differences obtained on Day 1 are maintained, but the difference in the gender factor increases between the groups. On the third day, it is only possible to differentiate the gender factor between the groups. Specifically, the differences between the different factors have been identified in each trial. Thus, we can highlight specific differences be-tween the groups as detailed below on supplementary data for males and females. In addition, differences between 3xTg-AD males and females were detected in the following trials (see Figure 6C and Table S4).

### 3.5. Biological Status: HPA Axis and Sarcopenia Index

**Figure 7:**
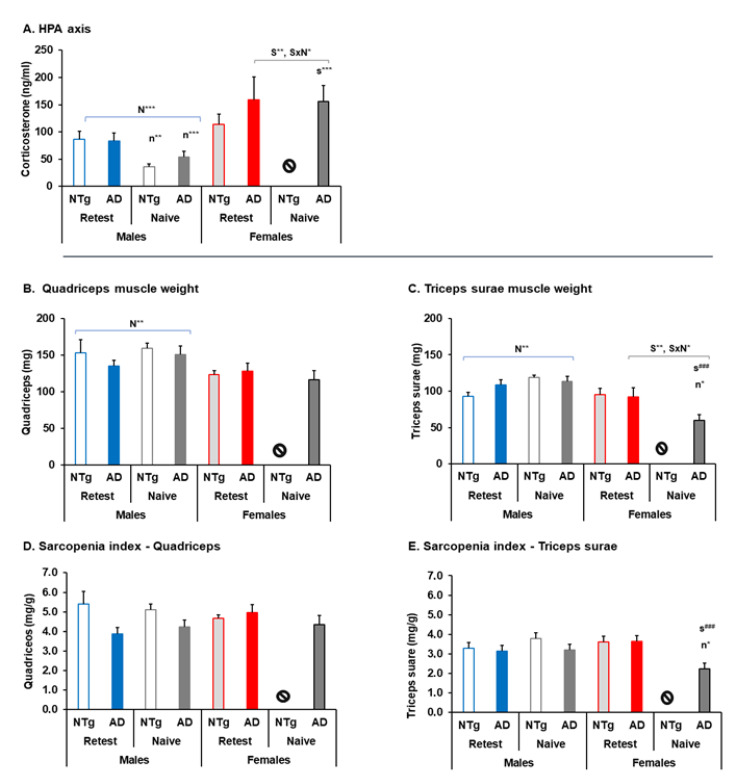
Biological status: HPA axis and sarcopenia index. (**A**) HPA axis, (**B**) quadriceps muscle weight, (**C**) triceps surae muscle, weight, (**D**) sarcopenia index—quadriceps, (**E**) sarcopenia index—triceps surae. Statistics: ANOVA, S, sex effect, S** *p* < 0.01**. N, naïve, naïve 16 months vs. re-test 16 months, N*** *p* < 0.001***, N** *p* < 0.01**. S×N, sex and naïve effects, S×N** *p* < 0.01**, S×N* *p* < 0.05*. Bonferroni *post hoc* test: s, sex; n: naïve, naïve 16 months vs. re-test 16 months; and # expressed sex differences between genotypes, s^###^, *p* < 0.001^###^ . The symbol ⦸ indicates the absence of the group, and m, month.

Higher differences were found in the corticosterone level in the re-test group compared to the naïve group (corticosterone, F (6, 70) = 9.817, *p* < 0.001 *post hoc:* male 3xTg-AD naïve vs. female 3xTg-AD naïve *p* < 0.001, male NTg naïve vs. male NTg re-test, *p* = 0.001). In addition, between group of males, the interaction of N had a lower level of corticosterone in the naïve group (N, F (1, 47) = 25.163, *p* < 0.001). In the 3xTg-AD group of animals, S effect and S×N interaction effects were differentiated (S, F (1, 42) = 16.456 *p* < 0.001. S×N, F (1, 42) = 4.243, *p* = 0.046), see Figure 7A.

The weight of the quadriceps and triceps sural muscles showed statistically significant differences (quadriceps, F (6, 70) = 3.203, *p* = 0.008. Triceps surae, F (6, 70) = 7.126, *p* < 0.001, *post hoc*: male naïve 3xTg-AD vs. female naïve 3xTg-AD, *p* < 0.001; female naïve 3xTg-AD vs. female re-test 3xTg-AD, *p* = 0.022). In addition, differences in the N effect were detected in male group, so, the muscle weight being greater in the naïve group in both muscles (quadriceps, N, F (1, 46) = 8.965, *p* = 0.005. Triceps surae, N, F (1, 46) = 7.267, *p* = 0.008). In the group of 3xTg-AD mice, differences in S and N were detected in the triceps surae muscle, the quadriceps muscle did not show significant differences in this analysis (triceps surae, S, F (1, 44) = 14.955, *p* < 0.001. S×N, F (1, 44) = 6.998, *p* = 0.012), see Figure 7B,C.

In the sarcopenia index, significant differences were observed in sarcopenia index- triceps surae (sarcopenia index, F (6, 70) = 3.158, *p* = 0.008, *post hoc*: male naïve 3xTg-AD vs. female naïve 3xTg-AD, *p* < 0.001; female naïve 3xTg-AD vs. female re-test 3xTg-AD, *p* = 0.022), see Figure 7E.

Furthermore, corticosterone levels were correlated with different variables, detecting a different correlation between males and females. In males, a negative correlation with the muscle weight of the quadriceps and triceps surae stands out, and a positive correlation with the variables, phenotype scoring system, frailty score, cadence and physical endurance on the first day (quadriceps, r^2^ = (-) 0.141, *p* = 0.008; triceps surae, r^2^ = (-) 0.098, *p* = 0.03).

**Figure 8:**
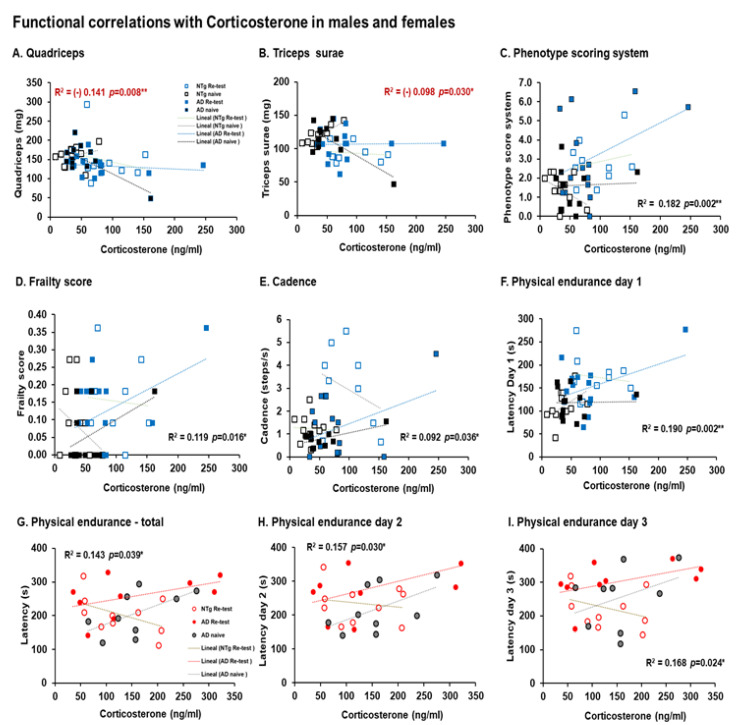
Functional corticosterone correlations in males and females. Pearson’s Correlations analysis of corticosterone in males and females. Meaningful, Pearson’s correlation in males between corticosterone and (**A**) quadriceps, (**B**) triceps surae, (**C**) phenotype scoring system, (**D**) frailty score, (**E**) cadence, and (**F**) physical endurance Day 1. Meaningful, Pearson’s correlation in females between corticosterone and (**G**) physical endurance—total, (**H**) physical endurance Day 2, (**I**) physical endurance Day 3. Statistics: Pearson r^2^, **p < 0.01, *p < 0.05.

Phenotype scoring system, r^2^ = 0.182, *p* = 0.002; frailty score, r^2^ = 0.119, *p* = 0.016; Cadence, r^2^ = 0.092, *p* = 0.036; Physical endurance day 1, r^2^ = 0.190, *p* = 0.002), see Figure 8A–F. In females, a positive correlation between corticosterone with performance in the rotarod on total, the second and third day were detected (physical endurance—total, r^2^ = 0.143, *p* = 0.039; physical endurance Day 2, r^2^ = 0.157, *p* = 0.03, physical endurance Day 3, r^2^ = 0.168, *p* = 0.024), see Figure 8G–I.

**Figure 9:**
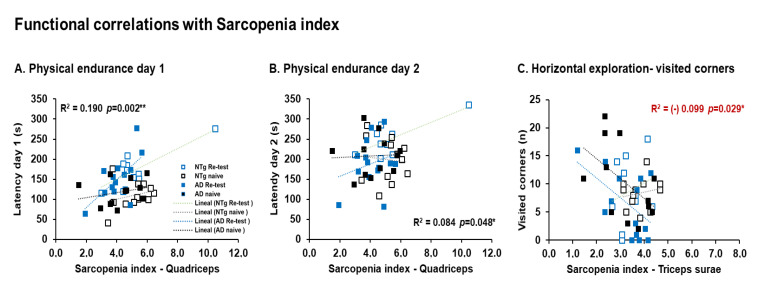
Functional correlations with sarcopenia index. Meaningful, Pearson’s correlations analysis of sarcopenia index. Meaningful, Pearson’s correlation between sarcopenia index quadriceps and (**A**) physical endurance Day 1, (**B**) physical endurance Day 2. Sarcopenia index, and triceps surae and (**C**) horizontal exploration—visited corners. Statistics: Pearson r^2^, **p < 0.01, *p < 0.05.

In different way, only in male, functional correlations with sarcopenia index were detected. Thus, sarcopenia index-quadriceps correlations with physical endurance Day 1 and Day 2 (sarcopenia index-quadriceps—physical endurance Day 1, r^2^ = 0.190, *p* = 0.002. sarcopenia index–quadriceps—physical endurance Day 2, r^2^ = 0.084, *p* = 0.048). In addition, sarcopenia index–triceps surae correlation with the number of horizontal explorations (visited corners) (sarcopenia index–triceps surae—corners, r^2^ = (−)0.099, *p* = 0.029), see Figure 9A–C. 

**Figure 10:**
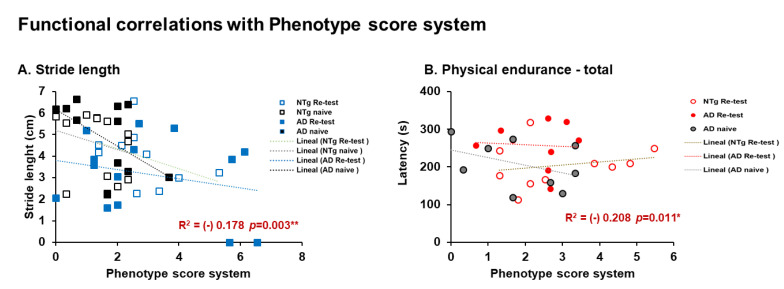
Functional correlations with phenotype score system. Pearson’s correlations analysis of phenotype score system. Meaningful, Pearson’s correlation between phenotype score system and (**A**) stride length in males, and (**B**) physical endurance—total in females. Statistics: Pearson r^2^, **p < 0.01, *p < 0.05.

On the other hand, males and females had a negative correlation between phenotype score system and functional variables. In the case of males, a negative correlation was detected between stride length and the phenotype scoring system (stride length—phenotype scoring system, r^2^ = (−) 0.178, *p* = 0.003). In females, a negative correlation is observed with physical endurance—total (phenotype scoring system, r^2^ = (−) 0.208, *p* = 0.011), see Figure 10A,B.

There is the summary of results in Table 2.

## 4. Discussion

Recently, we developed a battery of psychomotor tests that include gait, neophobia and exploration, muscle strength, motor learning, physical resistance, and frailty status [33]. The results, in males, indicated that 3xTg-AD mice exhibit a more significant functional impairment in the quantitative variables of gait and exploratory activity than age-matched NTg counterparts with normal aging. The presence of movement limitations and muscle weakness was determinant for the functional decline related to the stages of severity of the disease that worsened with age. In addition, we detected the presence of signs of physical frailty, which accompany the functional deterioration of these animals. The signs of sarcopenia were present in an advanced stage of AD [31,32]. Therefore, the present study was designed to investigate, for the first time, several aspects: (1) from a gender-medicine perspective, the impact of this functional impairment in 3xTg-AD females as compared to males; (2) the long-term effects of repeated test, either in longitudinal (the same set of animals at 12 and 16 months of age) or transversal (two different sets, pre-tested or naïve, at 16 months of age) designs, both in pathological and normal aging scenarios; (3) to include a phenotype of frailty and physical deterioration that may find a functional correlation with the biological status (HPA axis and sarcopenia), with nuances in male and female animals. 

### 4.1. Survival, Bodyweight, Phenotype Scoring System, Frailty Score, and Kyphosis

#### 4.1.1. Survival

The survival curves on the cohorts of 191 animals allowed us to record higher mortality in females, being the group of NTg females the one that presented the highest number of deaths between 8–12 months of age. Interestingly, only females under the longitudinal design survived and achieved 16 months of age, while the group of naïve NTg females perished before reaching that old age, suggesting that repeated testing might have some protective effects. These results agree with our previous reports in these colonies, where high mortality rates associated with increased frailty were reported in females, and NTg exhibited increased mortality from 12 months of age [42]. In the case of 3xTg-AD mice, females that reached old age were survivors who overcame the disease’s advanced neuropathological stages and exhibited lower behavioural differences with their NTg counterparts except for cognitive AD-hallmarks [47]. We have also described that, in male 3xTg-AD mice, an increase of mortality rates is associated with impairment in the neuro-immune-endocrine system compared to their females counterparts or the NTg genotype [48,49,50]. Noteworthy, we have recently reported survival bias and crosstalk between chronological and behavioral age in an APPswe model, where age- and genotype-sensitivity tests defined behavioral signatures in middle-aged, old, and long-lived mice with normal and AD-associated aging [51]. Therefore, the present work provides further evidence on sex and genotype-dependent differences in life expectancy and supports the key role of frailty and compensatory mechanisms as previously reported by our and other laboratories using different models of AD [29,49,50,51,52].

#### 4.1.2. Frailty

In the present work, the frailty results showed genotype differences between males, with NTg being the ones with the highest score. Only 12 of the 30 MCFI parameters were included as the incidence of the other indicators was very low or null. Kane and Brown [29] reported that 3xTg-AD male mice have a higher frailty index (FI) than NTg mice and 3xTg-AD females, and it was associated with their higher mortality ratios. Their study also indicated an increase in the frailty associated with age. On the other hand, in the present work, functional correlations in males found that their corticosterone levels correlated with frailty score and phenotype scoring system, both measures of functional decline. These results could indicate less deficit accumulation or functional capacity at the time of measurement in 3xTg-AD mice [53]. Therefore, it is plausible that other factors contribute to the survival/mortality of animals, and a complex multifactorial scenario be specific for each sex and biological age/stage of disease. In addition, in female C57BL/6 mice, greater frailty from 17 months of age with higher mortality at 26 months has been recently described in contrast to the non-fragile mice that reached 29 months of life [54]. These data have made it possible to identify that the prevalence of frailty in female mice increases throughout life and accurately predicts mortality [54]. Additionally, the animals’ bodyweight presented genotype differences that coincide with previous data [33] but the re-test decreased the weight in males, probably due to the training carried out at 12 months of age.

#### 4.1.3. Kyphosis

On the other hand, the severity of kyphosis was differentiated into postural and structural [31,32]. Here, genotype differences between males have been detected that corroborate previous reports, with greater severity in 3xTg-AD mice [31]. In females, here described for the first time, the severity of kyphosis increased with age and was more significant in the 3xTg-AD mice at 16 months in the re-test group, where the structural type predominates. The differences detected in males corroborate our other recent reports [32]. 

#### 4.1.4. Phenotype Scoring System

Kyphosis is also one of the scores included in the phenotype scoring system [39,40], which has recently been functionally differentiated by a severity classification that allows more information to be collected in contrast to other variables, such as those associated with gait and exploratory activity [32]. Thus, in the phenotype scoring system, we detected that kyphosis at 12 months of age was more significant in NTg of both sexes, a significance that was not reproduced at 16 months in these animals, which corroborates our differentiation of severity in the presence of kyphosis since the postural condition can be positionally modified. In addition, in the gait score, an increase in functional impairment was detected in 3xTg-AD males and females, which appears in the re-test group at 16 months. This variable makes it possible to discriminate a significant impairment of movement and exploratory activity since bizarre behaviours may occur that interfere with movement [31]. The deficits detected in the quantitative parameters of gait will be discussed in the following section. 

#### 4.1.5. Clasping

Finally, the presence of increased clasping in naïve 3xTg-AD mice at 16 months can also be highlighted. It was related to a more significant involved or progression of the disease [55,56]. The present results also suggest that repeated tests exerted protective effects in this respect. Lalonde [55] described brain regions and genes affecting limb-clasping responses. In the C57BL/6 strain, age-dependent locomotor deficits, including hindlimb clasping, are associated with a decreased number of dopaminergic neurons in aged mice, with reduced dopamine levels in the striatum [57]. Interestingly, alterations in the dopaminergic system described in 3xTg-AD mice and other AD models may also explain the presence of increased clasping.

### 4.2. Quantitative Parameters of Gait, and Neophobia and Exploration 

#### 4.2.1. Stride Length

Quantitative parameters in the gait analysis indicated that stride length was shorter in re-tested (16-month-old) male mice compared to age-matched naïve animals, and that this variable correlated with the gait phenotype score system. Interestingly, re-tested 3xTg-AD mice had the shortest stride length among the males compared to the naïve. In addition, differences in genotype and sex were observed at 12 and 16 months in the re-test group with greater stride length at 12 months in 3xTg-AD females and re-test in NTg males. In addition, the stride variability in females was lower than that of males, and the 3xTg-AD in all groups had the best performance, so their movement had more homogeneous steps throughout the trajectory. Previously, in our study in male 3xTg-AD mice of 6, 12, and 16 months of age, no differences in stride length or variability were detected, although a trend to increase stride length with age was observed in the case of 3xTg-AD mice while remained stable in the NTg genotype [31]. However, in another study at 6 months of age, increased stride length was reported in 3xTg-AD mice with no sex difference [58]. In addition, at 16 months of age, the gait of 3xTg-AD has been described as normal, without differences in genotype and sex [59,60]. According to the results, we propose that using the variability of the stride can help discriminate the trajectory of the movement during the gait analysis similar to humans where recently the variability was identified as a marker of cortical-cognitive dysfunction in AD patients [61,62]. 

#### 4.2.2. Speed

A significant decrease in speed in the male 3xTg-AD mice in all groups was observed. This decrease may be associated with a progressive functional decline in the 3xTg-AD male mice and coincides with the findings at 13 months of age we have previously reported [33]. Cadence had a lower performance in the 3xTg-AD males at 12 months of age. However, it increased at 16 months in the re-test group, differing from naïve at this age. Thus, cadence and speed are the variables with the highest sensitivity to discriminate genotypic differences in male mice and differentiate changes in gait attributable to pathological aging in the 3xTg-AD genotype. In the case of 3xTg-AD females, speed in-creases slightly in the 16-months re-test group and was higher than in males in all groups. At the clinical level, the identification of early changes in gait is of great relevance for identifying psychomotor disorders that in the case of AD may be related to the timing of steps and gait speed [63]. Additionally, corticosterone levels were positively correlated with a cadence in males.

#### 4.2.3. Neophobia and Exploration

The neophobia response, expressed as freezing, of 12 and 16-month-old naïve male mice was lower than in re-test mice in both genotypes, and statistically significant when contrasted with 16-month-old naïve mice. In females at 16 months of age, re-tested and naïve, a higher freezing was observed than in 16-month-old naïve females, albeit did not reach the statistical significance.

This neophobia emotional response is a characteristic of the 3xTg-AD model that is ac-companied by reduced immediate exploratory behaviour in a novel environment, as we first described in these animals in the open field test and the corner test already at the early ‘premorbid’ age of 2.5 months and worsened with the progress of the disease [64]. In addition, it corresponds to more sensitive ethological behaviours of the 3xTg-AD phenotype that has been reported in several other studies [31,33,42]. In addition, the horizontal exploratory activity did not report statistically significant differences. 

However, in the vertical exploratory activity (number and latency of rearings), differences between the re-test male mice at 16 months and the naïve of the same age were more statistically significant than the activity in naïve mice. In addition, the ratio (visited corners/rearings) in the re-test male mice of 12 and 16 months increased in the re-test but differed from the females at both ages, being lower in males at 12 months in both genotypes. At 16 months in re-test, NTg male’s ratio was high than NTg females, and in 3xTg-AD case, the ratio was increased in females 3xTg-AD. This decrease in activity over time, which is also observed in NTg mice, has been previously described as due to normal aging [64], with 3xTg-AD mice exhibiting less activity in most cases, which is attributed as a pathological trigger similar to BPSD that appear later in NTg mice due to normal aging [64]. In addition, in males was observed that correlated horizontal activity with triceps sural weight. 

### 4.3. Muscular Strength: Forelimb Grip Strength and Muscular Endurance—Hanger Test and Response to Gravity—Geotaxis

#### 4.3.1. Muscular Strength

Muscular strength is associated with global cognitive function in older people [65]. In addition, skeletal muscle mass index and physical performance (timed up and go test and grip strength) have decreased in older adults with AD [66]. Our results have not detected significant differences, although, at 12 months, it seems that females have a superior performance in grip strength and muscular endurance. Previously, we have reported that 13-month-old 3xTg-AD mice in natural isolation have preserved muscular strength [33] and that muscle strength and endurance would be associated with aging [31]. The laboratory of Brown also reported that at 6 months, 3xTg-AD mice have a deficit in grip strength [58], but at 16 months these results are not reproduced [59]. Additionally, the reduction in muscle weight and the appearance of sarcopenia may not yet be evident in the loss of muscle strength and resistance, or aging in this variable has greater importance than the distinction of the effects of the pathology in humans [67,68,69].

#### 4.3.2. Geotaxis

On the other hand, geotaxis showed differences between the males, with the 3xTg-AD re-test at 16 months being the ones that obtained a worse performance and the 3xTg-AD naïve females at 16 months. In addition, females take longer to pass the test, which is reflected in the differences in GxS in the 16-month-old re-test and naïve group. The usefulness of this test has been previously described [70]. Specifically, the geotaxis has allowed us to differentiate the animals’ postural positioning and balance strategies to pass the test and thus detect a possible functional deficit [31,33]. Therefore, 3xTg-AD re-test males and naïve females at 16 months show the most significant deterioration in this task.

### 4.4. Motor Performance: Learning and Physical Endurance—Rotarod

The motor performance showed superior performance in females of both genotypes. The motor learning tests and the number of trials reached the maximum values of the test in the re-test at 16 months. The increased performance may be due to pretraining done at 12 months, which can produce cognitive improvements with a long-term wheel of activity. In 16-month-old naïve 3xTg-AD males and females, lower latency and high number of trials were observed to achieve motor learning. Male 3xTg-AD mice have the most inferior performance in all tests.

As in motor learning, females have a high physical endurance. The 3xTg-AD females in the re-test group at 16 months achieved the highest performance over the male 3xTg-AD naïve and re-test, and female 3xTg-AD naïve 16-month-old females, and with similar performance to the NTg males of the same age. In addition, all groups increased their performance with training from Day 1 to Day 3, which is evident to a greater extent on the third day of training, and the effect of the re-test is observed at 16 months with an effect on different days for males and females, being in males on the first day of training and in females on the first and second day of training. Additionally, it was possible to distinguish the effect of aging in the naïve male NTg in contrast to the naïve at 12 months and re-test at 16 months. In addition, among the 3xTg-AD group, the sex differences between the 16-month-old re-test mice are distinguished from Day 1 to Day 3 of training. The 3xTg-AD males present the lowest performance among all groups, although with the training in the first day increased de physical endurance at 16 months in re-test group, on the following days, their performance is below 3xTg-AD naïve for 16 months.

The motor performance of 3xTg-AD mice has been reported in different studies. The performance in coordination and motor learning of 3xTg-AD mice has been highlighted over the performance of NTg mice, and these results are observable from 6 months and are reproduced at 16 months [58,59,60]. It has even been mentioned that 3xTg-AD females perform better than males at these ages [58,59]. In our laboratory, only reproduced the results of Stover et al. and Garvock-de Montbrun et. al. at 13 months, where the 3xTg-AD male mice presented a higher performance than the NTg, but in the latter, the weight factor interfered in the results [33]. Decreased motor function is also associated with aging, as reported in C57BL/6 mice of different ages [71,72,73]. In addition, we have differentiated the conceptualization of motor performance into motor learning—latency and motor learning—trials learning, since after physical exercise, the animals must manage to stay on a moving wheel in a coordinated manner. Consequently, in the first trials, physical endurance has a workload associated with an anaerobic exercise that progresses to aerobic exercise as the trials and their respective recovery times are replicated. In humans, the decrease in endurance exercise performance and its physiological determinants with aging appear to be mediated mainly by a reduction in the intensity (speed) and volume of exercise performed during training sessions [74]. Under this hypothesis, in their study, Pena et al. reported that 3xTg-AD mice improve their maximum latency in rotarod when subjected to aerobic exercise [75].

These results are accompanied by correlations with corticosterone levels and behave differently between males and females. In the case of males, corticosterone correlates positively with physical endurance on the first day of training, and in the case of females, it correlates positively with total physical endurance and physical endurance on the second and third days. On the other hand, a positive correlation was also detected in males between index-quadriceps sarcopenia and rotarod performance on the first and second days. A negative correlation was also detected between total physical endurance and the phenotype score system in females. Therefore, these interactions could explain the differences in performance between the groups studied.

### 4.5. Biological Status: HPA Axis and Sarcopenia Index

#### 4.5.1. Corticosterone

Corticosterone levels differed between groups due to sex and re-test factors, but not genotype. Males exhibited lower corticosterone levels in naïve mice of both genotypes, with similar levels between 3xTg-AD and NTg in the re-test group. On the contrary, in females, higher plasma corticosterone levels were observed in the 3xTg-AD re-test, and naïve females had similar levels that exceed the NTg re-test. It is also possible to distinguish that naïve 3xTg-AD females had higher levels than their male counterparts. The results agree with the sexual dimorphism reported by Muntsant et al., with higher plasma corticosterone levels in females [42], and also with plasma levels similar to the intervals described by Giménez-Llort et al. [76]. Additionally, corticosterone levels showed functional correlations with different variables depending on sex. In males, the correlation was inversely associated with the muscle mass of the quadriceps and triceps surae, and positively with frailty and gait cadence indicators. On the other hand, higher corticosterone levels correlated with higher performance on the first day of training in physical endurance. In females, the correlation with corticosterone was related to physical endurance performance with greater significance on the second and third training days. These results could indicate chronic stress if there is a long-term activation of the HPA axis in the case of females [77]. A report suggested that the combination of emotional and physical stress in a period of 5 h of exposure severely affected memory in NTg mice and increased the alterations in 3xTg-AD mice as a consequence of the reduction in the number dendritic spines and increase in the Aβ levels [50]. Additionally, the elevated corticosterone may precede cognitive impairments in genetically vulnerable 3xTg-AD females [78,79] and may, in turn, be related to frailty [80].

#### 4.5.2. Sarcopenia

Furthermore, we have observed that the quadriceps and triceps surae muscles have a greater weight in naïve male mice, whereas in 3xTg-AD females, a lower weight is observed in the triceps surae muscle with significant differences with the group of 3xTg-AD females and re-test and males of this genotype. These differences in naïve 3xTg-AD females are also observed in the sarcopenia index of the triceps surae muscle. In humans, sarcopenia is closely related to dementia, particularly AD, and may be involved in the pathophysiological process of AD [68,81]. On the other hand, poor muscle function but not reduced lean muscle mass drives the association of sarcopenia with cognitive decline in old age [67,82]. Sarcopenia, low grip strength, and slow walking speed were significantly associated with mild cognitive impairment in the community-dwelling elderly [80,83]. Therefore, our results can be helpful to study what occurs in human pathology through a translational approach to motor dysfunction at different levels of disability [31].

Moreover, in the case of males, the weight of the quadriceps and triceps surae muscles negatively correlated with plasma corticosterone levels. A positive correlation of the quadriceps sarcopenia index with physical endurance Days 1 and 2 was also found. In the case of the sural triceps sarcopenia index, it correlated negatively with the number of corners visited in the exploratory activity. These correlations were not found in females. 

Finally, the study’s limitations were given by the high mortality rate of NTg females that resulted in the lack of 16-month-old naïve group. Therefore, the genotype differences between 3xTg-AD and NTg females could not be contrasted. However, the analyses were carried out to detect the sex differences between the 3xTg-AD group. In future research, it would be interesting to compare the results of this study with NTg females since their functional profile may differ from males in physical or biological variables, such as in 3xTg-AD females.

## 5. Conclusions

From the results, it is possible to highlight that the high mortality rate in females, and among them that in the NTg group, was prevented in the group of females behaviorally assessed at 12 months of age, and these females were able to reach the age of 16 months completing the longitudinal design. In addition, higher corticosterone levels were detected in females and lower muscle weight of the triceps surae, which could indicate sarcopenia and alteration of the HPAaxis, which was more significant in the naive group at 16 months. Additionally, there were genotype-sensitive variables such as the phenotype scoring system, frailty and kyphosis in which the group of 3xTg-AD males showed physical deterioration. In turn, the motor learning and physical endurance variables were sensitive to re-testing, with 3xTg-AD females achieving the best performance when repeating the behavioral battery at 16 months. In addition, the females exhibited a better performance in gait, where their stride was homogeneous and straight. Additionally, females exhibited less severe scores in physical variables, such as kyphosis, which could explain males’ more significant deterioration in some motor tests. On the other hand, males showed deterioration in most of the variables studied. For their part, the correlations could explain the differences obtained between males and females, being positive in females between corticosterone and physical endurance, and the case of males between sarcopenia index and physical endurance as well as corticosterone with physical variables. The present results highlight the complexity of experimental scenarios in neurodegenerative diseases, such as Alzheimer’s disease, confirming not only the different impact of factors depending on genotype, sex, and age but their interplay with the methodological approach. They provide evidence that genotype, sex and age-dependent impact of behavioral assessment, as well as the repetition of behavioral tests, should not be underestimated. Conversely, and most importantly, the ability of behavioral assessment and repeated tests to modify the behavioral outputs indicates that they could be considered functional trainings that modify survival, anxiety, and functional profile (physical endurance and motor learning) of old male and female 3xTg-AD mice and also NTg mice counterparts with normal aging.

## Figures and Tables

**Table 1:** Phenotype scoring system.

Phenotype Scoring System		Naïve 12-Month-Old			Re-Test 16-Month-Old			Naïve 16-Month-Old			Statistics
		Males	Females	*p*-Value	Males	Females	*p*-Value	Males	Females	*p*-Value	
**Clasping score**	**NTg**	0.52 ± 0.16	0.43 ± 0.08	n.s.	0.67 ± 0.20	0.69 ± 0.17	n.s.	0.25 ± 0.06	NR	g*	n.s.
	**AD**	0.56 ± 0.17	0.62 ± 0.24		0.79 ± 0.19	0.30 ± 0.15		0.67 ± 0.18 *	0.26 ± 0.12		
**Ledge score**	**NTg**	0.40 ± 0.15	0.48 ± 0.14	n.s.	0.60 ± 0.09	0.68 ± 0.11	n.s.	0.31 ± 0.12	NR	n.s.	R**
	**AD**	0.25 ± 0.08	0.48 ± 0.13		0.58 ± 0.16	0.51 ± 0.07		0.22 ± 0.09	0.41 ± 0.14		
**Gait score**	**NTg**	0.21 ± 0.15	0.33 ± 0.12	G×S**	0.20 ± 0.13	0.38 ± 0.15	G×S**	-	NR	s^#^	G*, R**, r^&&^, r^$$^
	**AD**	0.26 ± 0.10	-		0.64 ± 0.13^&&^	-		0.03 ± 0.03	0.44 ± 0.24		
**Kyphosis score**	**NTg**	1.07 ± 0.16	1.0 ± 0.25	G**	1.30 ± 0.15	1.38 ± 0.15	n.s.	0.72 ± 0.21, s^$$^	NR	n.s.	G*, R***, r^&^
	**AD**	0.30 ± 0.13	0.43 ± 0.20		0.95 ± 0.26	1.37 ± 0.27 ^&^		0.47 ± 0.17	0.67 ± 0.28		
**Total score**	**NTg**	2.21 ± 0.37	2.24 ± 0.39	G*	2.77 ± 0.37	3.14 ± 0.45	n.s.	1.28 ± 0.23	NR	n.s.	R***, r^&^ G*
	**AD**	1.37 ± 0.30	1.52 ± 0.33		2.96 ± 0.58 ^&^	2.18 ± 0.32		1.39 ± 0.28	1.78 ± 0.44		

Statistics: ANOVA, G, genotype effect, G** *p* < 0.01, *p* < 0.05, n.s. *p* > 0.05. R, Re-test effect, R*** *p* < 0.001***, R** *p* < 0.01**, *p* < 0.05*, n.s. *p* > 0.05. G×S, genotype and sex interaction effect, G×S** *p* < 0.01**, *p* < 0.05*, n.s. *p* > 0.05. Bonferroni post hoc test: g, genotype, g* *p* < 0.05*; s, sex; $ expressed genotype differences between sex, s^$$^ *p* < 0.01^$$^; & expressed differences between re-test groups, r^&&^ *p* < 0.01^&&^, *p* < 0.05^&^; $ expressed genotype differences between re-test group, r^&^ *p* < 0.05^&^, and r^$$^, between sex differences. NR indicates the absence of the group.

**Table 2:** Summary of results.

	Genotype Factor (G)	Sex Factor (S)	Re-Test Factor (R)	Naïve Factor (N)
Phenotype scoring system	🡅 deficits 3xTg-AD group 🡅 deterioration in 3xTg-AD males in the total score			
Frailty	🡅 3xTg-AD males at 16 m in the re-test			
Kyphosis	🡅 3xTg-AD males increased the severity in the re-test at 16 m			
Quantitative parameters of gait	Speed: 🡅 3xTg-AD males at 12 m, and 🡇 in the re-test at 16 m 🡇 16 m naïve NTg and 3xTg-AD Cadence: 🡇 3xTg-AD males at 12 m and 16 m Re-test 🡇 3xTg-AD and NTg Re-test group at 12 and 16 m 🡇 Naïve 16 m NTg and 3xTg-AD males had a lower cadence than age-matched re-tests	Speed: 🡅 Re-test and naïve 16 m 3xTg-AD females Variability of stride length: 🡇 Re-test and naïve 16 m 3xTg-AD females	Stride length: 🡇 Re-test 3xTg-AD males at 12 m Cadence: 🡇 Naïve 16 m males 3xTg-AD and NTg 🡇3xTg-AD male in all groups	Stride length: 🡅 Naïve 3xTg-AD and NTg at 16 m
Exploration and neophobia		Exploratory activity (ratio): 🡅 NTg and 3xTg-AD females at 12 m		Freezing: 🡇 Naïve 3xTg-AD and NTg males at 16 m Vertical exploratory activity: 🡅 Naïve 3xTg-AD and NTg males at 16 m Exploratory activity (ratio): 🡅 Naïve 3xTg-AD and NTg males at 16 m 🡅 Naïve 3xTg-AD females at 16 m
Geotaxis		🡅 Naïve 3xTg-AD females at 16 m	🡅 Re-test 3xTg-AD in the re-test group compared to their performance at 12 m.	
Motor learning	🡅 Latency 3xTg-AD males Re-test at 16 m	🡅 Latency and trials females at 12 m in both genotypes. 🡅 Females at 16 m re-test group	🡅 Latency re-test 3xTg-AD males at 16 m 🡅 Latency re-test 3xTg-AD females at 16 m 🡅 N trials among males in re-test group 🡇 N trials among females in re-test group	🡇 Latency naïve 3xTg-AD male and females at 16 m
Physical Endurance	🡇 3xTg-AD males at 12 m and 16 m in the re-test group. 🡇 Day 2, 3xTg-AD males in all groups 🡇 Day 3, 3xTg-AD males at 12 m	🡅 3xTg-AD females at 12 m and 16 m 🡅 Day 2–3, 3xTg-AD females at 12 m and 16 m	🡅 NTg re-test males at 16 m 🡅 Re-test at 16 m in all group in 2nd and 3rd training days 🡅 Re-test at 16 m male groups in 1st training day 🡅 Re-test at 16 m female group in 1st and 2nd day	🡅 16 m naïve 3xTg-AD males than 3xTg-AD Re-test at this age.
HPA axis		🡅 3xTg-AD re-test at 16 m 🡅 Naïve females at 16 m		🡇 Naïve Re-test males at 16 m 🡅 Naïve females at 16 m
Sarcopenia index		🡇 Triceps surae and sarcopenia index naïve 3xTg-AD females		🡅 Quadriceps and triceps sura muscles naïve males Re-test at 16 m. 🡇 Triceps surae and sarcopenia index naïve 3xTg-AD females re-test females at 16 m
Survival	High mortality, mostly among NTg female mice, rescued in longitudinal designs			
Correlation’s interactions	In males, negative correlations between corticosterone and quadriceps, triceps surae; and positive correlations between corticosterone and phenotype score system, frailty score, cadence, and physical endurance Day 1. Females, positives correlated between corticosterone and physical endurance-total, physical endurance Days 2 and 3. Positive correlations in males were detected between sarcopenia index-quadriceps and physical endurance on Days 1 and 2. In females, negative correlations were detected between sarcopenia index–triceps and horizontal activity. Negative correlations in males were identified between phenotype score system and stride length, and in females’ phenotype score system and physical endurance—total.			

According to the factors, genotype (G), sex (S), re-test (R) and naïve (N), a summary of the main results of this study is presented. It also includes the correlation’s interactions. The symbol 🡅 indicates increase, 🡇 indicates decreases, and m, month.

## Data Availability

Not applicable.

## Supplementary Materials

The following supporting information can be downloaded at: https://www.mdpi.com/article/10.3390/biomedicines10050973/s1, Table S1: Physical performance in males and females Naïve 12-month-old 3xTg-AD and NTg mice; Table S2: Physical performance in males and female 3xTg-AD and NTg after Re-test to 16m; Table S3: Physical performance in males and females Naïve 16-month-old 3xTg-AD and NTg mice; Table S4: Statistics Figure 5C.
