# Supplementary material for: Impact of Behavioral Assessment and Re-Test as Functional Trainings That Modify Survival, Anxiety and Functional Profile (Physical Endurance and Motor Learning) of Old Male and Female 3xTg-AD Mice and NTg Mice with Normal Aging
Source: Biomedicines. 2022 Apr 22;10(5):973. doi: 10.3390/biomedicines10050973 (PMC9138863; doi:10.3390/biomedicines10050973)
Supplement: Supplementary file 1 [file biomedicines-10-00973-s001.zip › biomedicines-1618096-supplementary.pdf]

biomedicines

MDPI

Supplementary data

Table S1. Physical performance in males and females Naïve 12-month-old 3xTg-AD and NTg mice.

Functional Profile	Males		Females		Statistics
	NTg mice n=13 (Mean ± SEM)	3xTg-AD mice n=19 (Mean ± SEM)	NTg mice n=17 (Mean ± SEM)	3xTg-AD mice n=14 (Mean ± SEM)	ANOVA
**Bodyweight**	29.31 ± 0.56	35.67 ± 1.79 p = **0.039**$^{\$}$	27.15 ± 1.12	33.93 ± 2.14 p = **0.024**$^{\$}$	***G, p < 0.001****
**Quantitative parameters of gait - Gait**					
Stride length (*cm*)	5.00 ± 0.36	4.25 ± 0.46	4.28 ± 0.35	5.19 ± 0.26	***GxS, p = 0.037****
Variability of stride length (%)	33.60 ± 3.00	30.47 ± 3.59	30.28 ± 3.00	19.53 ± 2.98	***S, p = 0.015****
Speed (*cm/s*)	10.78 ± 2.10	5,34 ± 1.01 ***p = 0.018***$^{\$}$	8.10 ± 1.87	6.71 ± 1.10	***GxS, p = 0.033**** ***S, p = 0.015****
Cadence (*steps/s*)	3.76 ± 0.62	1.86 ± 0.35 ***p = 0.007***$^{\$\$}$	3.28 ± 0.72	1.52 ± 0.26	***G, p = 0.002*****
**Exploration and Neophobia – Corner test**					
Horizontal activity (*n corners*)	9.15 ± 0.85	8.74 ± 1.32	9.13 ± 0.98	9.58 ± 1.43	***n.s.***
Vertical activity (*n rearings*)	2.85 ± 0.89	2.16 ± 0.59	2.13 ± 0.53	2.36 ± 0.54	***n.s.***
Rearing latency (*s*)	39.46 ± 6.15	32.31 ± 5.30	31.54 ± 4.74	29.37 ±5.26	***n.s.***
Ratio (corners/rearings)	2.02 ± 0.73	2.53 ± 0.69	3.95 ± 0.93	5.38 ± 1.26	***S, p = 0.015****
Freezing (movement latency, *s*)	0.94 ± 0.49	5.69 ± 3.21	4.64 ± 3.73	1.34 ± 0.65	***n.s.***
**Response to gravity- Geotaxis**					
Geotaxis *(latency, s)*	5.77 ± 0.65	4.61 ± 0.64	4.61 ± 0.39	6.21 ± 0.60	***GxS****
**Motor performance - Rotarod**					
Motor learning *(latency, s)*	29.23 ± 6.58	18.79 ± 4.55	45.53 ± 5.73	32.00 ± 6.79	***S****
Motor learning - *(trials learning)*	2.54 ± 0.42	3.37 ± 0.44	1.82 ± 0.35	1.86 ± 0.27 ***p = 0.010***$^{\#}$	***S, p = 0.004*****
Physical endurance *(total latency, s)*	216.35 ± 15.28	144.03 ± 13.83 ***p = 0.001***$^{\$\$}$	182.30 ± 9.99	200.76 ± 13.50 ***p = 0.014***$^{\#}$	***GxS, p = 0.001*****$^{\#}$
Day 1 *(total latency, s)*	141.68 ± 15.25	91.46 ± 9.05 ***p = 0.020***$^{\$}$	116.49 ± 10.19	122.40 ± 12.19	***GxS, p = 0.019****
Day 2 *(total latency, s)*	245.85 ± 18.40	151.75 ± 18.47 ***p = 0.001***$^{\$\$}$	206.91 ± 13.31	213.79 ± 17.78	***G, p = 0.012*,*** ***GxS, p = 0.004*****
Day 3 *(total latency, s)*	261.53 ± 17.45	188.89 ± 17.26 ***p = 0.014***$^{\$}$	223.50 ± 12.91	266.10 ± 17.71 ***p = 0.006***$^{\#\#}$	***GxS, p = 0.001*****

G, Genotype effect, G*** p < 0.001, G** *p* < 0.01, G* *p* < 0.05, *n.s.* *p* > 0,05. S, Sex effect, S*** *p* < 0.001, S** *p* < 0.01, S* *p* < 0.05, *n.s.* *p* > 0,05. GxS, genotype and sex interaction effects, GxS*** *p* < 0.01, GxS** *p* < 0.05, GxS* *p* < 0.05, *n.s.* p > 0,05. Bonferroni *post hoc* test: g, genotype; s, sex; and $ expressed genotype differences between sex, and # expressed sex differences between genotypes.

Table S2. Physical performance in males and female 3xTg-AD and NTg after Re-test to 16m.

Functional Profile	Males		Females		Statistics	
	**NTg mice**<br>***n*=10**<br>**(Mean ± SEM)**	**3xTg-AD mice**<br>***n*=14**<br>**(Mean ± SEM)**	**NTg mice**<br>***n*=12**<br>**(Mean ± SEM)**	**3xTg-AD mice**<br>***n*=9**<br>**(Mean ± SEM)**	**ANOVA**<br>**(Retest)**	**MRA**<br>**(12m vs. 16m)**
**Bodyweight**	29.09 ± 0.40	33.63 ± 1.55<br>p = **0.035**$^{\$}$	25.65 ± 0.78	26.37 ± 0.80<br>p < **0.001**$^{\#}$	*G, p = 0.026**	*G, p = 0.041**<br>S, *p* = **0.003****
S, p < **0.001****						
**Quantitative parameters of gait - Gait**						
Stride length (*cm*)	3.61 ± 0.41	2.96 ± 0.49<br>***p* = 0.023**$^{\$}$	3.03 ± 0.49	4.63 ± 0.49	***GxS, p = 0.017****	***n.s.***
Variability of stride length (%)	22.18 ± 4.23	30.50 ± 5.53	27.61 ± 6.09	18.53 ± 2.15	***n.s.***	***S, p = 0.026****
Speed (*cm/s*)	6.85 ± 1.33	2.69 ± 0.79<br>***p = 0.026***$^{\$}$	5.48 ± 1.31	7.36 ± 1.35	***GxS, p = 0.042****	***n.s.***
Cadence (*steps/s*)	3.04 ± 0.53	2.24 ± 0.36<br>***p* = 0.004**$^{\$}$	2.83 ± 0.67	2.39 ± 0.51	***n.s.***	***G, p = 0.011****
**Exploration and Neophobia – Corner test**						
Horizontal activity (*n corners*)	8.70 ± 1.88	6.92 ± 1.48	6.82 ± 1.88	11.56 ± 2.70	***n.s.***	***n.s.***
Vertical activity (*n corners*)	1.50 ± 0.87	0.38 ± 0.30	0.82 ± 0.64	1.44 ± 0.56	***n.s.***	***n.s.***
Rearing latency (*s*)	38.11 ± 7.77	53.38 ± 2.83	51.48 ± 5.78	36.70 ± 7.82	***n.s.***	***n.s.***
Ratio (corners/rearings)	2.95 ± 1.82	1.19 ± 0.68	0.54 ± 0.42	5.02 ± 2.02	***GxS, p = 0.037****<br>***GxS, p = 0.008***$^{\#\#}$	***GxS, p = 0.007*****
Freezing (movement latency, *s*)	7.67 ± 5.87	10.60 ± 6.88	11.19 ± 7.28	0.88 ± 0.58	***n.s.***	***n.s.***
**Response to gravity- Geotaxis**						
Geotaxis *(latency, s)*	4.95 ± 0.83	7.75 ± 1.03<br>***p = 0.042***^{r}	6.61 ± 1.02	5.87 ± 1.01	***GxS, p = 0.012****	***n.s.***
**Motor performance - Rotarod**						
Motor learning *(latency, s)*	39.00 ± 8.65	56.64 ± 3.13<br>***p < 0.001***^{rrr}<br>***p = 0.025***$^{\$}$	60.00 ± 0.00	60.00 ± 0.00	***G, p = 0.042*,***<br>***GxS, p = 0.042****	***S, p = 0.021****
Motor learning *(trials learning)*	1.60 ± 0.26	1.15 ± 0.10	1.00 ± 0.00	1.00 ± 0.00<br>***p = 0.019***$^{\#}$	***S, p = 0.07*****	***S, p = 0.024****
Physical endurance *(latency, s)*	228.43 ± 10.27	184.04 ± 15.60	209.64 ± 18.58	257.94 ± 20.07<br>***p = 0.010***$^{\#}$	***GxS, p = 0.005*****	***GxS, p = 0.005*****
Day 1 *(total latency, s)*	179.48 ± 14.89	141.9 ± 11.29	176.63 ± 18.66	188.15 ± 23.17	***n.s.***	***n.s.***
Day 2 *(total latency, s)*	250.09 ± 14.13	189.44 ± 17.42	222.13 ± 21.83	282.93 ± 27.78<br>***p = 0.008***$^{\#\#}$	***GxS, p = 0.003*****	***GxS, p = 0.004*****
Day 3 *(total latency, s)*	255.72 ± 11.86	220.9 ± 20.71	229.87 ± 20.49	302.76 ± 20.27<br>***p = 0.017***$^{\#}$	***GxS, p = 0.006*****	***GxS, p = 0.003*****

MRA, naïve 12m - retest 16m, ANOVA. G, Genotype effect, G*** $p < 0.01$, G** $p < 0.05$, G* $p < 0.05$, *n.s.* $p > 0{,}05$. S, Sex eefct, S*** $p < 0.01$, S** $p < 0.05$, S* $p < 0.05$, *n.s.* $p > 0{,}05$. GxS, genotype and sex interaction effects, GxS*** $p < 0.01$, GxS** $p < 0.05$, GxS* $p < 0.05$, *n.s.* $p > 0{,}05$. Bonferroni *post hoc* test: g, genotype; s, sex; and $ expressed genotype differences between sex, and # expressed sex differences between genotypes, and r expressed re-test differences between 12m and 16m in the same group.

Table S3. Physical performance in males and females Naïve 16-month-old 3xTg-AD and NTg mice.

Functional Profile	Males		Females		Statistics	
	**NTg mice**<br>***n*=12**<br>**(Mean ± SEM)**	**3xTg-AD mice**<br>***n*=12**<br>**(Mean ± SEM)**	**NTg mice**<br>**-**<br>**(Mean ± SEM)**	**3xTg-AD mice**<br>***n*=9**<br>**(Mean ± SEM)**	**ANOVA**<br>**Naïve 16m**	**ANOVA**<br>**Naïve vs. Retest**
**Bodyweight**<br>**s, $p < 0.001^{\#\#\#}$**	31.53 ± 0.90	36.44 ± 1.81<br>p = **$0.023^{\$}$**	-	26.90 ± 0.40<br>**$p < 0.001^{\#}$**	**G, p = 0.001****	**R, $p = 0.006^{**}$**<br>**g , $p = 0.005^{\$\$}$**<br>**s, $p < 0.001^{\#\#\#}$**
**Quantitative parameters of gait - Gait**						
Stride length (*cm*)	4.59 ± 0.42	4.80 ± 0.50	-	4.51 ± 0.58	***n.s.***	***N, $p = 0.023^{r}$***<br>***NxS, $p = 0.047^{\#}$***
Variability of stride length (%)	25.53 ± 2.71	25.79 ± 3.92	-	20.25 ± 4.46<br>***$p = 0.026^{\#}$***	***n.s.***	***S, $p = 0.026^{\#}$***
Speed (*cm/s*)	3.54 ± 0.91	2.93 ±0.47	-	5.72 ± 1.83	***n.s.***	***G, $p = 0.022^{\$}$***<br>***S, $p = 0.014^{\#}$***
Cadence (*steps/s*)	1.24 ± 0.16<br>***$p = 0.037^{r}$***	0.80 ± 0.09	-	1.72 ± 0.67	***n.s.***	***G, $p = 0.001^{\$\$}$***<br>***R, $p = 0.016^{r}$***
**Exploration and Neophobia – Corner test**						
Horizontal activity (*n corners*)	8.33 ± 0.80	10.25 ± 1.95	-	10.11 ± 2.62	***n.s.***	***n.s.***
Vertical activity (*n rearings*)	2.58 ± 0.86	3.75 ± 1.11	-	1.33 ± 0.50	***n.s.***	***N, $p = 0.005^{\$\$}$***<br>***N, $p = 0.044^{\#}$***<br>***NxS, $p = 0.023^{\#}$***
Rearing latency (*s*)	27.44 ± 7.23	32.69 ± 7.15	-	36.30 ± 7.90	***n.s.***	***N, $p = 0.022^{\$}$***
Ratio (corners/rearings)	1.85 ± 0.51	1.44 ± 0.41	-	4.35 ± 2.22	***n.s.***	***N, $p = 0.039^{\$}$***
Freezing (movement latency, *s*)	0.09 ± 0.09	1.04 ± 0.64	-	14.05 ± 8.70	***n.s.***	***N, $p = 0.013^{\$}$***
**Response to gravity- Geotaxis**						
Geotaxis *(latency, s)*	5.47 ± 0.99	4.24 ± 0.49<br>***$p = 0.041^{r}$***	-	8.21 ± 1.14	***n.s.***	***GxR, $p = 0.009^{**}$***<br>***GxS, $p = 0.002^{**}$***
**Motor performance – Rotarod**						
Motor learning *(latency, s)*	44.08 ± 6.55	29.08 ± 6.99<br>***$p = 0.007^{nn}$***	-	30.33 ± 8.34<br>***p = 0.026n***<br>***$p < 0.001^{\#\#\#}$***	***n.s.***	***GxR, $p = 0.014^{\$}$***
Motor learning *(trials learning)*	1.58 ± 0.22	2.08 ± 0.28<br>***$p = 0.012^{n}$***	-	3.00 ± 0.85<br>***p = 0.022n***<br>***$p < 0.001^{\#\#\#}$***	***n.s.***	***R, $p = 0.042^{\$}$***<br>***GxR, $p = 0.042^{*}$***
Physical endurance *(total latency, s)*	177.62 ± 12.01<br>***$p < 0.001^{aaa}$***<br>***$p < 0.001^{nnn}$***	189.27 ± 11.56<br>***$p = 0.001^{nn}$***	-	206.58 ± 21.47<br>***p = 0.001nn***<br>***$p = 0.001^{aa}$***	***n.s.***	***R, $p < 0.001^{\$\$\$}$***<br>***R, $p < 0.001^{\#\#\#}$***<br>***S, $p = 0.004^{\#\#}$***
Day 1 *(total latency, s)*	110.09 ± 9.66	118.65 ± 9.31<br>***$p < 0.001^{aaa}$***<br>***$p = 0.025^{n}$***	-	147.44 ± 24.10	***n.s.***	***R, $p = 0.006^{\$\$}$***<br>***S, $p = 0.022^{\#}$***
Day 2 *(total latency, s)*	199,23 ± 16.02	208.43 ± 14.56 ***p***<br>***$< 0.004^{aa}$***	-	217.17 ± 23.20	***n.s.***	***S, $p = 0.017^{\#}$***
Day 3 *(total latency, s)*	223.54 ± 17.86<br>***$p = 0.009^{aa}$***	240.73 ± 15.31	-	255.13 ± 30.47<br>***$p = 0.013^{n}$***	***n.s.***	***R, $p = 0.001^{\$\$}$***<br>***R, $p = 0.001^{\#\#}$***

***p* = 0.008**[nn]	**S, *p* = 0.011**[#]

G, Genotype effect, G* $p < 0.05$, *n.s.* $p > 0,05$. S, Sex effect, S*** $p < 0.01$, S** $p < 0.05$, *n.s.* $p > 0,05$. Bonferroni *post hoc* test: s, sex; and # expressed sex differences between genotypes; $ expressed genotype differences between sex, and # expressed sex differences between genotypes; a expressed age differences in each group; r expressed re-test differences between 12m and 16m in the same group, and n expressed naive differences between re-test 16m and naïve16m in the same group.

Table S4: Statistics Figure 5C

Figure 5C.1<br>Males: MRA -ANOVA, Day-by-day1, F (5,74) = 6.779, $p < 0.001$, *post hoc* NTg naïve 12m *vs.* 3xTg-AD naïve 12m $p = 0.023$, NTg naïve 16m *vs.* NTg re-test 16m $p = 0.012$, 3xTg-AD naïve 12m *vs.* 3xTg-AD re-test 16m $p = 0.002$. Genotype differences, MRA-ANOVA, F (1,76) = 4.638, $p = 0.034$. Re-test differences, MRA-ANOVA, F (1,76) = 20.040, $p < 0.001$.
Figure 5C.2<br>Males: MRA -ANOVA, Day-by-day 2, F (5,73) = 5.078, $p < 0.001$, *post hoc* naïve 12-month-old NTg *vs.* naïve 3xTg-AD 12m $p = 0.001$. Genotype differences, MRA-ANOVA, F (1,75) = 11.412, $p = 0.01$.
Figure 5C.3<br>Males: MRA -ANOVA, Day-by-day 3, F (5,73) = 2.733, $p = 0.026$, *post hoc* naïve NTg 12m *vs.* naïve 3xTg-AD 12m $p = 0.028$. Genotype differences, MRA-ANOVA, F (1,75) = 4.480, $p = 0.038$.
Figure 5C.4<br>Males: MRA -ANOVA, Trial-by-trial, F (5,73) = 5.211, $p < 0.001$. Genotype differences, MRA-ANOVA, F (1,75) = 8.815, $p = 0.004$. Re-test differences, MRA-ANOVA, F (1,75) = 4.692, $p = 0.033$.
Figure 5C.5<br>Females: MRA -ANOVA, Trial-by-trial, F (4,65) = 3.137, $p = 0.021$, *post hoc* NTg naïve 12m *vs.* 3xTg-AD re-test 16m $p = 0.009$.
Figure 5C.6<br>Females: MRA -ANOVA, Day-by-day 1, F (4,56) = 4.438, $p = 0.03$, *post hoc* NTg naïve 12m *vs.* NTg re-test 16m $p = 0.028$, NTg naïve 12m vs. re-test 3xTg-AD 16m $p = 0.025$.
Figure 5C.7<br>Females: MRA -ANOVA, Day-by-day 3, F (4,56) = 2.701, $p = 0.040$.
Figure 6C.8<br>3xRg-AD: MRA -ANOVA, Trial-by-trial, Trial by trial: MRA-ANOVA, F (5,71) = 5.586 , $p < 0.001$. Sex differences, MRA-ANOVA, F (1,73) = 15.213, $p < 0.001$. Re-test differences, MRA-ANOVA, F (1,73) = 7.873, $p = 0.006$.
Figure 5C.9<br>3xTg-AD: MRA -ANOVA, Day-by-day 1, F (5,71) = 5.424, $p < 0.001$, *post hoc* females re-test 16m vs. naïve males 12m $p < 0.001$, re-test females 16m vs. 16m naïve males $p = 0.042$. Sex differences: MRA-ANOVA, F (1,73) = 6.537, $p = 0.013$. Re-test differences: MRA-ANOVA, F (1,73) = 16.327, $p < 0.001$.
Figure 5C.10<br>3xTg-AD: MRA -ANOVA, Day-by-day 2, F (5,71) = 4.694, $p = 0.001$, *post hoc* females re-test 16m vs. naïve males 12m $p < 0.001$, re-test females 16m vs. 16m naïve males $p = 0.036$. Sex differences: MRA-ANOVA, F (1,73) = 14.001, $p < 0.001$. Re-test differences: MRA-ANOVA, F (1,73) = 5.371, $p = 0.023$.
Figure 5C.11<br>3xTg-AD: MRA -ANOVA, Day-by-day 3, F (5,71) = 4.077, $p = 0.003$, *post hoc* females re-test 16m vs. naïve males 12m $p = 0.002$. Sex differences: MRA-ANOVA, F (1,73) = 13.723, $p < 0.001$.
Figure 5C.12<br>Males - Day 1: T1, ANOVA, F (5,74) = 4.931, $p = 0.001$, *post hoc* naïve 3xTg-AD 12m vs. 3xTg-AD re-test 16m $p = 0.004$; Re-test differences, ANOVA, F (1,78) = 18.084, $p < 0.001$. T2, ANOVA, F (5,74) = 4.260, $p = 0.002$, *post hoc* naïve 3xTg-AD 12m vs. 3xTg-AD re-test 16m $p = 0.007$; Re-test differences, ANOVA, F (1,78) = 18.307, $p < 0.001$. T3, ANOVA, F (5,74) = 3.981, $p = 0.003$, *post hoc* naïve 3xTg-AD 12m vs. 3xTg-AD re-test 16m $p = 0.011$; Re-test differences, ANOVA, F (1,78) = 12.376, $p = 0.001$. T4, ANOVA, F (5,74) = 5.452, $p < 0.001$, *post hoc* NTg re-test 16m vs. naïve NTg 16m $p = 0.003$, 3xTg-AD naïve 16m vs. NTg naïve 16m $p = 0.005$; Genotype differences, ANOVA, F (1,78) = 11.578, $p = 0.001$; Re-test differences, ANOVA, F (1,78) = 12.228, $p = 0.001$. T5, ANOVA, F (5,74) = 4.868, $p = 0.001$, *post hoc* NTg re-test 16m vs. naïve NTg 16m $p = 0.016$, 3xTg-AD naïve 12m vs. 3xTg-AD re-test 16m $p = 0.025$; Re-test differences, ANOVA, F (1,78) = 16.546, $p < 0.001$. T6, ANOVA, F (5,74) = 5.916, $p < 0.001$, *post hoc* 3xTg-AD naïve 12m vs. NTg

naïve 12m p = 0.002, 3xTg-AD naïve 12m vs. 3xTg-AD re-test 16m p = 0.006; Genotype differences, ANOVA F (1,78) = 8.355, p = 0.005; Re-test differences, ANOVA, F (1,78) = 11.347, p = 0.001.
Figure 5C.13<br>Males - Day 2: T1, ANOVA, F (5,74) = 2.963, p = 0.027, *post hoc* 3xTg-AD naïve 12m vs. NTg naïve 12m p = 0.017; Genotype differences, ANOVA, F (1,78) = 4.168, p = 0.045. T3, ANOVA, F (5,74) = 5.098, *post hoc* 3xTg-AD naïve 12m vs. NTg naïve 12m p = 0.002; Genotype differences, ANOVA, F (1,78) = 8.601, p = 0.004. T4, ANOVA, F (5,74) = 5.807, p < 0.001, *post hoc* 3xTg-AD naïve 12m vs. NTg naïve 12m p = 0.001; Geno-type differences, ANOVA, F (1,78) = 12.959, p = 0.001. T5, ANOVA, F (5,74) = 8.099, p < 0.001, *post hoc* 3xTg-AD naïve 12m vs. NTg naïve 12m p < 0.001, 3xTg-AD re-test 16m vs. NTg re-test 16m p = 0.033, 3xTg-AD naïve 12m vs. 3xTg-AD naïve 16m p = 0.014; Genotype differences, ANOVA, F (1,78) = 17.675, p < 0.001. T6, Genotype differences, ANOVA, F (1,78) = 6.282, p = 0.014.
Figure 5C.14<br>Males - Day 3: T1, ANOVA, F (5,74) = 3.075, p = 0.014; Genotype differences, ANOVA, F (1,78) = 6.477, p = 0.013; Re-test differences, ANOVA, F (1,78) = 6.616, p = 0.012. T4, ANOVA, F (5,74) = 2.617, p = 0.031, *post hoc* 3xTg-AD naïve 12m vs. NTg naïve 12m p = 0.026. T5, Genotype differences, ANOVA, F (1,78) = 5.066, p = 0.027.
Figure 5C.15<br>Females – Day 1: T1, ANOVA, F (4,56) = 3.996, p = 0.006, *post hoc* NTg naïve 12m vs. NTg re-test 16m p = 0.045. T2, ANOVA, F (4,56) = 4.477, p = 0.003, *post hoc* NTg naïve 12m vs. NTg re-test 16m p = 0.015. T4, ANOVA, F (4,56) = 3.729, p = 0.009, *post hoc* NTg naïve 12m vs. re-test NTg 16m p = 0.026. T5, ANOVA, F (4,56) = 2.809, p = 0.034. T6, ANOVA, F (4,56) = 4.909, p = 0.002, *post hoc* NTg naïve 12m vs. NTg re-test 16m p = 0.008.
Figure 5C.16<br>Females - Day 2: T1, ANOVA, F (4,56) = 2.588, p = 0.046, *post hoc* 3xTg-AD naïve 12m vs. 3xTg-ASD re-test 16m p = 0.028. T3, ANOVA, F (4,56) = 3.272, p = 0.018.
Figure 5C.17<br>Females - Day 3: T3, ANOVA, F (4,56) = 2.606, p = 0.045. T4, ANOVA, F (4,56) = 2.988, p = 0.026. T5, ANOVA, F (4,56) = 3.025, p = 0.025, *post hoc* 3xTg-AD retest 16m vs. NTg re-test 16m p = 0.045. T6, ANOVA, F (4,56) = 2.731, p = 0.038, see figure 5C.
Figure 5C.18<br>3xTg-AD - Day 1, T1, ANOVA, F (5,71) = 4.431, p = 0.001, *post hoc* female re-test 16m vs. Male naïve 12m p = 0.002; Re-test differences, ANAOVA, F (1,76) = 16.401, p < 0.001. T2, ANOVA, F (5,71) = 4.372, p = 0.002, *post hoc* female re-test 16m vs. male naïve 12m p = 0.004, female re-test 16m vs. male naïve 16m p = 0.050; Re-test differences, ANOVA, F (1,76) = 15.013, p < 0.001. T3, ANOVA, F (5,71) = 4.144, p = 0.002, *post hoc* female re-test 16m vs. male naïve 12m p = 0.002; Re-test differences, ANOVA, F (1,76) = 11.434, p = 0.001. T4, ANOVA, F (5,71) = 2.572, p = 0.034, *post hoc* female re-test 16m vs. male naïve 12m p = 0.035; Sex differences, ANOVA, F (1,76) = 5.752, p = 0.019; Re-test differences, ANOVA, F (1,76) = 5.725, p = 0.019. T5, ANOVA, F (1,76) = 4.794, p = 0.001, *post hoc* female re-test 16m vs. male naïve 12m p = 0.042; Sex differences, ANOVA, F (1,76) = 5.952, p = 0.017; Re-test differences, ANOVA, F (1,76) = 13.912, p < 0.001. T6, ANOVA, F (5,71) = 5.723, p < 0.001, *post hoc* female re-test 16m vs. male naïve 12m p < 0.001; Sex differences, ANOVA, F (1,76) = 7.737, p = 0.007; Re-test differences, ANOVA, F (1,76) = 15.056, p < 0.001.
Figure 5C.19<br>3xTg-AD - Day 2: T1, ANOVA, F (5,71) = 3.104, p = 0.014, *post hoc* female re-test 16m vs. male naïve 12m p = 0.005; Re-test differences, ANOVA, F (1,76) = 9.296, p = 0.003. T2, Sex differences, ANOVA, F (1,76) = 6.607, p = 0.012; Re-test differences, ANOVA, F (1,76) = 4.183, p = 0.044. T3, ANOVA, F (5,71) = 5.092, p < 0.001, *post hoc* female re-test 16mvs. male naïve 12m p < 0.001, female re-test 16m vs. male re-test 16m p = 0.004; Sex differences, ANOVA, F (1,76) = 16.280, p < 0.001; Re-test differences, ANOVA, F (1,76) = 4.017, p = 0.049; SxR differences, ANOVA, F (1,76) = 4.912, p = 0.030. T4, ANOVA, F (5,71) = 4.043, p = 0.003, *post hoc* female re-test 16m vs. male naïve 12m p = 0.001; Sex differences, ANOVA, F (1,76) = 13.637, p < 0.001. T5, ANOVA, F (5,71) = 5.511, p < 0.001, *post hoc* female naïve 12m vs. male naïve 12m p = 0.015; Sex differences, ANOVA, F (1,76) = 13.849, p < 0.001. T6, ANOVA, F (5,71) = 3.529, p = 0.007; Sex differences, ANOVA, F (1,76) = 13.549, p < 0.001.
Figure 5C.20<br>3xTg-AD - Day 3: T1, Sex differences, ANOVA, F (1,76) = 6.160, p = 0.015. T2, ANOVA, F (1,76) = 6.384, p = 0.014. T3, ANOVA, F (5,71) = 3.942, p = 0.003, *post hoc* female naïve 12m vs. male naïve 12m p = 0.048, female re-test 16m vs. male naïve 12m p = 0.007; Sex differences, ANOVA, F (1,76) = 13.021, p = 0.001. T4, ANOVA, F (5,71) = 3.789, p = 0.004, *post hoc* female naïve 12m vs. male naïve 12m p = 0.045, female re-test 16m vs. male naïve 12m p = 0.004; Sex differences, ANOVA, F (1,76) = 10.172, p = 0.002. T5, ANOVA, F (5,71) = 5.281, p < 0.001, *post hoc* female naïve 12m vs. male naïve 12m p = 0.011, female re-test 16m vs. male naïve 12m p = 0.012; Sex differences, ANOVA, F (1,76) = 18.229, p < 0.001. T6, ANOVA, F (5,71) = 3.822, p = 0.004, *post hoc* female re-test 16m vs. male naïve 12m p =0.003; Sex differences, ANOVA, F (1,76) = 14.803, p < 0.001.
